# Further engineering of *R. toruloides* for the production of terpenes from lignocellulosic biomass

**DOI:** 10.1186/s13068-021-01950-w

**Published:** 2021-04-21

**Authors:** James Kirby, Gina M. Geiselman, Junko Yaegashi, Joonhoon Kim, Xun Zhuang, Mary Bao Tran-Gyamfi, Jan-Philip Prahl, Eric R. Sundstrom, Yuqian Gao, Nathalie Munoz, Kristin E. Burnum-Johnson, Veronica T. Benites, Edward E. K. Baidoo, Anna Fuhrmann, Katharina Seibel, Bobbie-Jo M. Webb-Robertson, Jeremy Zucker, Carrie D. Nicora, Deepti Tanjore, Jon K. Magnuson, Jeffrey M. Skerker, John M. Gladden

**Affiliations:** 1 Department of Energy, Agile BioFoundry, Emeryville, CA 94608 USA; 2 Department of Biomass Science and Conversion Technology, Sandia National Laboratories, Livermore, CA 94550 USA; 3 Joint BioEnergy Institute, Lawrence Berkeley National Laboratory, Emeryville, CA 94608 USA; 4 Chemical and Biological Processing Group, Pacific Northwest National Laboratory, Richland, WA 99354 USA; 5 Advanced Biofuels and Bioproducts Process Development Unit, Lawrence Berkeley National Laboratory, Emeryville, CA 94608 USA; 6 Biological Systems and Engineering Division, Lawrence Berkeley National Laboratory, Berkeley, CA 94720 USA; 7 Environmental Genomics and Systems Biology Division, Lawrence Berkeley National Laboratory, Berkeley, CA 94720 USA; 8 QB3-Berkeley, University of California, Berkeley, CA 94704 USA; 9 The Environmental Molecular Sciences Laboratory, Richland, WA 99354 USA; 10 Biological Sciences Division, Pacific Northwest National Laboratory, Richland, WA 99354 USA

**Keywords:** *Rhodotorula*, Mevalonate pathway, Isoprenoids, Metabolic engineering, Α-bisabolene, Eucalyptol, 1,8-Cineole

## Abstract

**Background:**

Mitigation of climate change requires that new routes for the production of fuels and chemicals be as oil-independent as possible. The microbial conversion of lignocellulosic feedstocks into terpene-based biofuels and bioproducts represents one such route. This work builds upon previous demonstrations that the single-celled carotenogenic basidiomycete, *Rhodosporidium toruloides*, is a promising host for the production of terpenes from lignocellulosic hydrolysates.

**Results:**

This study focuses on the optimization of production of the monoterpene 1,8-cineole and the sesquiterpene α-bisabolene in *R. toruloides*. The α-bisabolene titer attained in *R. toruloides* was found to be proportional to the copy number of the bisabolene synthase (BIS) expression cassette, which in turn influenced the expression level of several native mevalonate pathway genes. The addition of more copies of BIS under a stronger promoter resulted in production of α-bisabolene at 2.2 g/L from lignocellulosic hydrolysate in a 2-L fermenter. Production of 1,8-cineole was found to be limited by availability of the precursor geranylgeranyl pyrophosphate (GPP) and expression of an appropriate GPP synthase increased the monoterpene titer fourfold to 143 mg/L at bench scale. Targeted mevalonate pathway metabolite analysis suggested that 3-hydroxy-3-methyl-glutaryl-coenzyme A reductase (HMGR), mevalonate kinase (MK) and phosphomevalonate kinase (PMK) may be pathway bottlenecks are were therefore selected as targets for overexpression. Expression of HMGR, MK, and PMK orthologs and growth in an optimized lignocellulosic hydrolysate medium increased the 1,8-cineole titer an additional tenfold to 1.4 g/L. Expression of the same mevalonate pathway genes did not have as large an impact on α-bisabolene production, although the final titer was higher at 2.6 g/L. Furthermore, mevalonate pathway intermediates accumulated in the mevalonate-engineered strains, suggesting room for further improvement.

**Conclusions:**

This work brings *R. toruloides* closer to being able to make industrially relevant quantities of terpene from lignocellulosic biomass.

**Supplementary Information:**

The online version contains supplementary material available at 10.1186/s13068-021-01950-w.

## Background

Terpenes, or isoprenoids, constitute one of the largest groups of natural products, playing a variety of diverse roles from steroid hormone biosynthesis in humans to pollinator attraction in plants [1]. Besides the many applications that terpenes play in health and nutrition, they have also attracted a considerable amount of attention as candidates for renewable fuels and bioproducts. Monoterpenes such as pinene and 1,8-cineole show promise as potential precursors to renewable jet fuels [2]. A number of C_15_ sesquiterpenes such as α-bisabolene [3] and farnesene [4] can function as diesel fuels, following reduction to their respective alkanes by hydrogenation. Other terpenes such as the C_20_ diterpene, *ent*-kaurene may provide bio-based replacements for petrochemicals with a variety of industrial applications [5].

This work builds upon previous efforts to produce 1,8-cineole and α-bisabolene in the single-celled basidiomycete, *Rhodosporidium toruloides*. This fungus has previously been identified as an attractive host for production of biofuels and bioproducts from lignocellulosic biomass, in part due to its ability to co-metabolize multiple carbon sources, including C_5_ and C_6_ sugars and aromatic compounds found in lignocellulosic hydrolysates [6].

1,8-Cineole, or eucalyptol, is the major constituent of eucalyptus oil, but is also produced by several other plants and an endophytic fungus, *Hypoxylon sp.* [7, 8]. Research into the use of eucalyptus oil as a fuel goes back more than three decades, when its suitability for addition to gasoline–ethanol blends was examined. More recently, representatives of the aviation industry announced plans to expand its scope to use in jet fuel, prompting an increased interest in microbial production of 1,8-cineole from lignocellulosic feedstocks [9]. Monoterpene titers in microbial hosts are typically lower than titers achieved for other classes of terpene. Although titers of around 0.5 g/L have been reached in *Escherichia coli* for the monoterpenes limonene [10], 1,8-cineole, and linalool [11], much lower titers are usually observed in fungal hosts such as *Saccharomyces cerevisiae* [12]. Previous efforts to produce 1,8-cineole from lignocellulosic biomass in *R. toruloides* by expression of *HYP3* from *Hypoxylon *sp*. E7406B* resulted in a titer of 35 mg/L [2].

Research on microbial production of terpenes has been more heavily weighted towards sesquiterpenes, which are generally less toxic to microbes than monoterpenes and leave the cell more readily compared to larger terpenes such as carotenoids. Production of α-bisabolene was reported in *S. cerevisiae* and *R. toruloides* at titers of 900 mg/L and 680 mg/L, respectively [3, 6], with the latter produced from lignocellulosic hydrolysate. Somewhat surprisingly, the diterpene *ent*-kaurene was produced at higher titers (1.4 g/L) from lignocellulosic hydrolysate in *R. toruloides*, perhaps owing to the fact that the diterpene precursor, geranylgeranyl pyrophosphate (GGPP) was enhanced by expression of a heterologous GGPP synthase in the engineered strain [5]. In contrast, production of α-bisabolene and 1,8-cineole has thus far relied on native levels of their respective precursors, farnesyl pyrophosphate (FPP) and geranyl pyrophosphate (GPP). GPP in particular is produced in limited amounts in *R. toruloides* as the longer prenyl phosphate precursor FPP is generated in a single step from the mevalonate pathway C_5_ products, isopentenyl pyrophosphate (IPP) and dimethylallyl pyrophosphate (DMAPP). It therefore seems likely that 1,8-cineole titers in *R. toruloides* are limited by GPP levels and that production of all classes of terpenes may benefit from engineering to increase metabolic flux to IPP and DMAPP. Successive cycles of design, build, test, and learn (DBTL) were applied to explore these limitations to high-level terpene production in *R. toruloides*.

## Results and discussion

### Enhancing α-bisabolene titer in *R. toruloides*

**Fig. 1:**
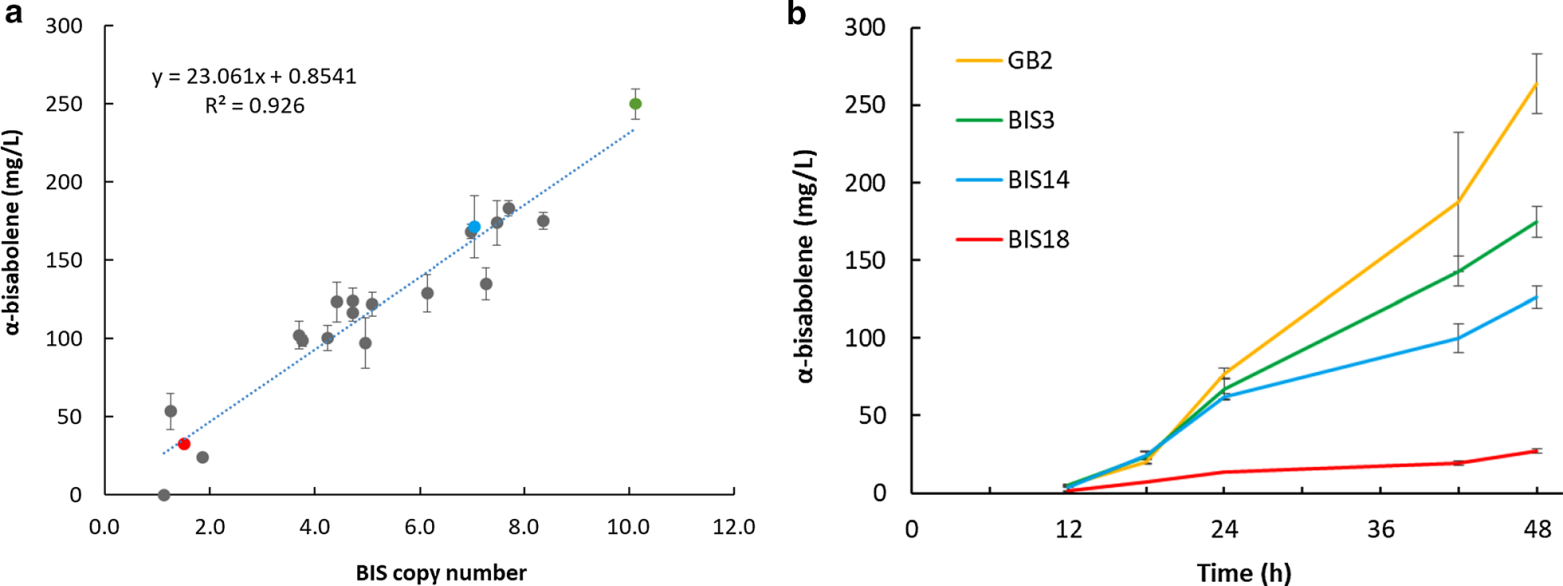
Engineering the production of α-bisabolene in *R. toruloides*. **a** Correlation between P_*GAPDH*_-BIS copy number and α-bisabolene titer at day 8 in SD medium containing 20 g/L glucose. **b** Production of α-bisabolene in SD medium containing 10 g/L glucose by three strains harboring the P_*GAPDH*_-BIS cassette at various copy numbers (shown as points of corresponding color in A) and strain GB2, built by insertion of 6 copies of P_*ANT*_-BIS into strain BIS3. BIS, α-bisabolene synthase from *Abies grandis*; P_*GAPDH*_, *R. toruloides* glyceraldehyde 3-phosphate dehydrogenase promoter; P_*ANT*_, *R. toruloides* adenine nucleotide translocase promoter

Previous work on engineering *R. toruloides* for production of α-bisabolene indicated that flux through the native mevalonate pathway is relatively high in this species. A strain modified only by insertion of a heterologous α-bisabolene synthase gene (BIS) from *Abies grandis* under control of the native *R. toruloides GAPDH* (glyceraldehyde 3-phosphate dehydrogenase) promoter into WT *R. toruloides* achieved α-bisabolene titer of 294 mg/L in a defined medium containing 2% (w/v) glucose and 680 mg/L in a 2-L bioreactor fed with corn stover hydrolysate [6]. Since heterologous DNA was introduced into *R. toruloides* by ATMT, which results in DNA integration at random loci with a variable copy number, it was of interest to investigate the correlation between copy number and α-bisabolene titer for a range of P_*GAPDH*_-BIS transformants. A good correlation (*R*^2^ = 0.93, *p* = 1e−11) exists between α-bisabolene titer and P_*GAPDH*_-BIS copy number for the 20 strains examined and, since a plateau did not appear to have been reached, it seemed likely that BIS expression remained a limiting factor for α-bisabolene production (Fig. 1a). The linearity of this correlation also suggests that insertion locus may be less important than copy number in determining the level of heterologous gene expression. To test the hypothesis that the α-bisabolene titer is still limited by BIS gene expression, the highest-titer P_*GAPDH*_-BIS strain, BIS3, was selected for addition of a second expression cassette consisting of BIS under control of the native *R. toruloides* ANT (adenine nucleotide translocase) promoter [15]. This resulted in strain, GB2, which produced 1.5-fold more α-bisabolene than the parent strain, BIS3, and contained 6 copies of the P_*ANT*_-BIS cassette in addition to the original 10 copies of the P_*GAPDH*_-BIS cassette in BIS3 (Fig. 1b).

### Scale up of α-bisabolene production in lignocellulosic hydrolysate

**Fig. 2:**
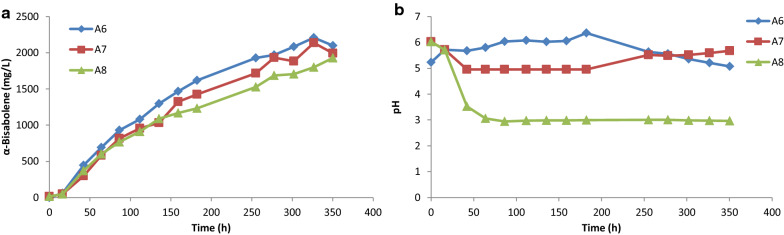
Production of α-bisabolene at 2-L scale. Strain GB2 was grown in medium containing DMR-EH supplemented with either 10 g/L yeast extract (bioreactor A6) or 5 g/L ammonium sulfate (bioreactors A7 and A8). Bioreactors A6 and A7 received a sodium hydroxide feed if the pH dropped below 5.0, while reactor A8 received no pH control. α-bisabolene titer (**a**) and pH (**b**) were monitored for 14 days. DMR-EH, Lignocellulosic hydrolysate prepared from corn stover by deacetylation and mechanical refining followed by enzymatic hydrolysis

*R. toruloides* has been identified as a promising host for production of renewable biofuels and bioproducts because it can efficiently utilize mixed carbon sources and tolerate potential growth inhibitors often found in lignocellulosic hydrolysates [6]. We therefore wanted to determine how the new high BIS-copy GB2 strain performs when grown on a lignocellulosic hydrolysate derived from corn stover (DMR-EH) for α-bisabolene production [13]. Growth and productivity were measured in 2-L bioreactors and the impact of nitrogen source and pH were investigated. Two of the bioreactors (A6 and A7, respectively) were used to compare two nitrogen sources supplementing the DMR-EH base medium: complex (10 g/L yeast extract) and defined (5 g/L ammonium sulfate). The medium supplemented with defined nitrogen also contained 100 µM iron sulfate and 100 mM potassium phosphate (pH 6.0). Medium pH was maintained above 5.0 during growth in these two bioreactors, while a third culture (A8) was grown in the defined nitrogen DMR-EH medium with no pH control. Although the pH in the third bioreactor dropped significantly (~ pH 3) over the course of the run, the final α-bisabolene titer, 1.9 g/L, was close to that of the pH-controlled cultures (Fig. 2). The two pH-controlled cultures grown on defined nitrogen and complex nitrogen also reached similar α-bisabolene titers, 2.1 and 2.2 g/L, respectively. The pH in the culture supplemented with yeast extract rose slightly to around pH 6.0 during the first half of the fermentation, only dropping as low as pH 5.0 by the end of the fermentation, while the culture supplemented with ammonium sulfate required an adjustment to keep the pH above 5.0 from days 2 to 8 (Fig. 2b).

### Transcriptomics and proteomics analysis of α-bisabolene producing strains

**Fig. 3:**
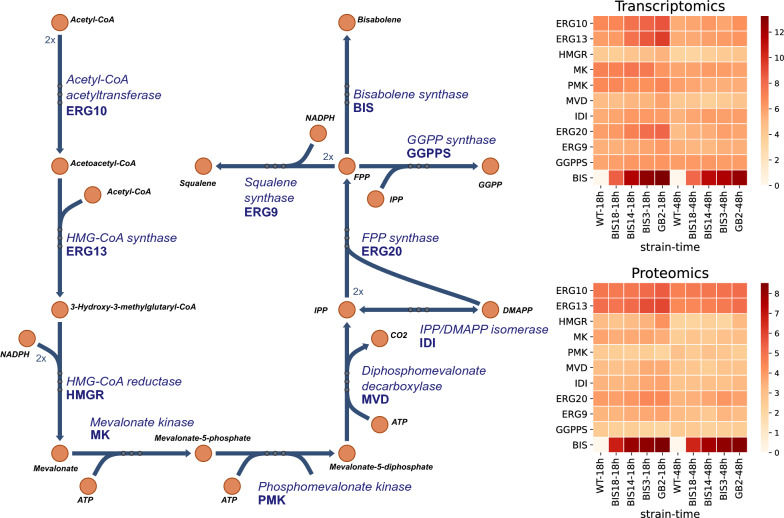
Change in mevalonate pathway gene expression in response to α-bisabolene production in *R. toruloides*. Transcriptomics and proteomics data is shown for five *R. toruloides* strains (WT, BIS18, BIS14, BIS3, and GB2) sampled following 18 h and 48 h of growth in SD medium containing 10 g/L glucose. The average of log2 transformed FPKM values and spectral counts for three replicates are used for transcriptomics and proteomics, respectively. FPKM, Fragments Per Kilobase of transcript per Million mapped reads

Generation of strains that produce different tiers of α-bisabolene provides an opportunity to understand how *R. toruloides* respond to the diversion of carbon flux toward a heterologous product and to identify potential targets for metabolic engineering. To examine this response on a systems level, wild-type *R. toruloides* and four strains that produced α-bisabolene at various titers (Fig. 1b) were selected for global proteomic and transcriptomic analysis. Growth and sugar consumption were monitored in SD medium containing 10 g/L glucose and samples taken at 18 and 48 h were used for omics analysis (Additional file 1: Fig. S1). Both protein and transcript data indicate that BIS is one of the most highly expressed genes in the highest-titer strain, GB2, harboring both P_*GAPDH*_-BIS and P_*ANT*_-BIS (Fig. 3 and Additional file 1: Table S1). Comparing the strains, BIS transcript and protein levels increase in parallel with the increase in copy number and α-bisabolene titer, surpassing expression levels of two of the strongest native genes, *ANT* and *TEF1* (translation elongation factor 1α), in most cases (Additional file 1: Table S1). Interestingly, expression of the first two enzymes of the mevalonate pathway, acetyl-CoA acetyltransferase (ERG10) and 3-hydroxy-3-methylglutaryl-CoA (HMG-CoA) synthase (ERG13), also increase in proportion to α-bisabolene production, suggesting that the cell induces regulation to increase metabolic flux into the mevalonate pathway to accommodate the diversion of carbon away from native mevalonate pathway products to α-bisabolene. The same is true of the native FPP synthase (ERG20), which supplies the precursor to α-bisabolene and as well as being a key control point in ergosterol biosynthesis. The expression of other pathway genes underwent more modest changes in expression, even decreasing slightly in GB2, suggesting that they may be good targets for metabolic engineering to enhance flux through the mevalonate pathway. For example, transcript and protein levels for mevalonate kinase (MK) decreased slightly while phosphomevalonate kinase (PMK) changed little in strain GB2 compared to WT (Fig. 3 and Additional file 1: Table S2).

Also notable was the upregulation of ATP citrate lyase (ACLY), acetyl-CoA carboxylase (ACC1), and fatty acid synthase (FAS2) in GB2, relative to WT, possibly responding to changes in acetyl-CoA levels. An upregulation of sterol metabolism genes such as squalene monooxygenase (ERG1), lanosterol synthase (ERG7), and lanosterol 14-alpha-demethylase (ERG11) was also observed (Additional file 1: Table S2).

### Enhancing 1,8-cineole production in *R. toruloides* by increasing GPP supply

In this study, we attempt to optimize production of both α-bisabolene and 1,8-cineole. Before attempting to optimize the mevalonate pathway for both terpenes, we first wanted to ensure that there were sufficient pools of the pyrophosphate precursors for these two products. While the α-bisabolene precursor FPP is a central metabolite in the ergosterol pathway, 1,8-cineole is made from GPP, which previous work indicates is significantly more limited in *R. toruloides* [2]. Similar to *S. cerevisiae* and other fungi, *R. toruloides* lacks a dedicated GPP synthase (GPPS), and FPP is made directly from the C_5_ precursors, IPP and DMAPP by the enzyme ERG20. Therefore, the initial strategy for engineering higher 1,8-cineole titers focused on optimizing and balancing expression levels of the two terminal enzymes: GPPS and 1,8-cineole synthase. Engineering a high monoterpene titer requires a balance between GPPS and terpene synthase activities that provides sufficient flux to accumulate the target product while avoiding growth-inhibitory levels of GPP [11, 27]. Promoters for these synthases were selected from native *R. toruloides* genes. Three promoters, *ANT*, *GAPDH*, and *TEF1* were selected based on their relative strength, constitutive expression profiles, and utility in prior work [2, 3, 5, 15]. P_*ANT*_ and P_*GAPDH*_ were used to express *HYP3* from *Hypoxylon sp. E7406B*, encoding a 1,8-cineole synthase previously identified as a promising enzyme for monoterpene production in *R. toruloides *[2]. P_*TEF1*_ was used to drive candidate GPPSs, several of which are FPP synthases containing mutations that alter the prenyl phosphate product chain length specificity in favor of GPP. Genes were transformed into *R. toruloides* by ATMT in either stepwise fashion (gene stacking) or by the use of single, combined constructs. Following each transformation event, up to 40 strains were screened for 1,8-cineole production and the best strains were retested in triplicate in YPD_10_ medium. The highest-titer strain resulting from each transformation was selected for comparison and further strain engineering.

**Fig. 4:**
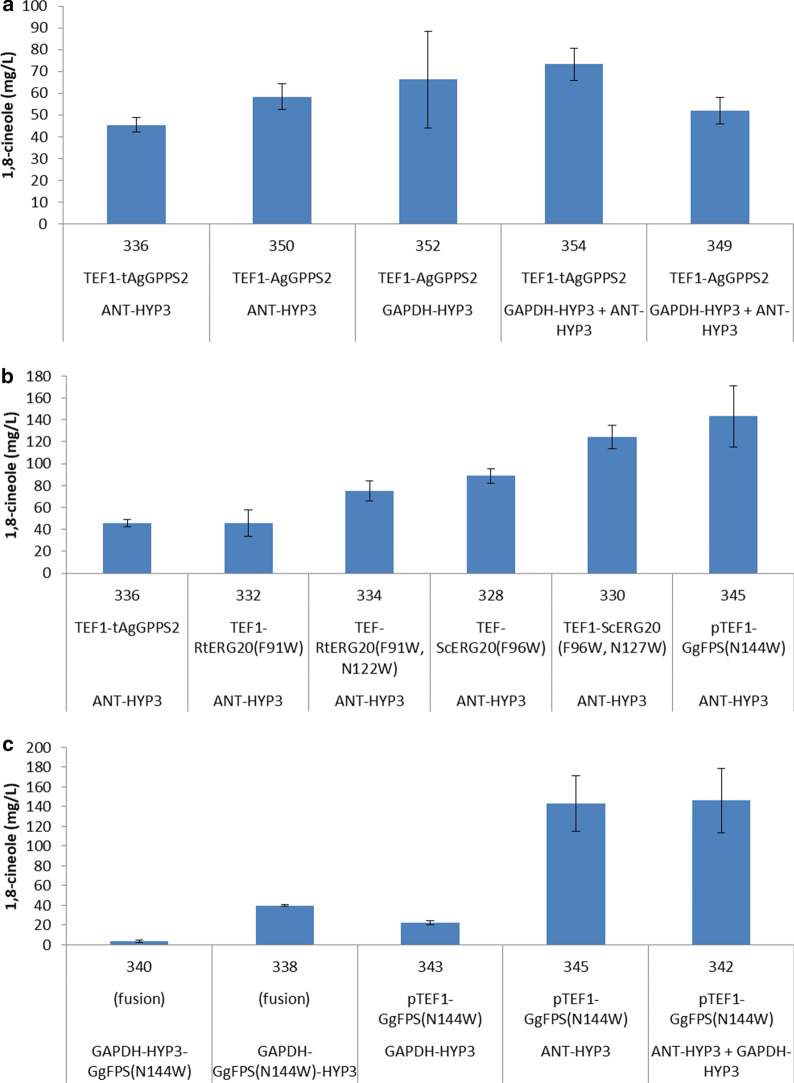
Engineering 1,8-cineole production in *R. toruloides*. Strains are described in Table 1. **a** Various combinations of *HYP3* and *AgGPPS2*. **b** Different GPP synthases combined with P_*ANT*_-*HYP3*. **c** Assorted strategies for expression of *GgFPS(N144W)* and *HYP3*, individually and as fusion proteins. Titers were measured following 5 days of cultivation in culture tubes containing YPD_10_ medium. HYP3, 1,8-cineole synthase from *Hypoxylon sp. E7406B; AgGPPS2,* GPP synthase from *A. grandis*; P_*ANT*_, *R. toruloides* adenine nucleotide translocase promoter; *GgFPS,* FPP synthase from *Gallus gallus*

Various configurations of *HYP3* were combined with a GPPS from *A. grandis* with the N-terminal plastid transit peptide removed (*tAgGPPS2*) or not (*AgGPPS2*), under control of the *TEF1* promoter (Fig. 4a). Each of these strains demonstrated some improvement over the previously published 1,8-cineole titer of 35 mg/L, suggesting that GPP synthesis is limiting to some extent [2]. Combining P_*TEF1*_-*AgGPPS2* with P_*ANT*_-*HYP3* (strain 350) resulted in a 1,8-cineole titer of 58 mg/L and switching the *HYP3* promoter from P_*ANT*_ to P_*GAPDH*_ did not change the titer significantly (strain 352, producing 66 mg/L 1,8-cineole). A comparison of strains harboring both P_*GAPDH*_-*HYP3* and P_*ANT*_-*HYP3* with either P_*TEF1*_-t*AgGPPS2* or P_*TEF1*_-*AgGPPS2* indicates that the truncated GPPS (strain 354, 73 mg/L 1,8-cineole) performed slightly better than the full-length version (strain 349, 52 mg/L 1,8-cineole).

Success with engineering monoterpene production has been reported by the use of FPP synthases harboring mutations that alter the substrate-binding pocket such that prenyl phosphate chain length elongation beyond C_10_ (GPP) is significantly reduced [11, 28–30]. Strains were constructed to compare various mutant FPP synthases, all under control of P_*TEF1*_ and combined with P_*ANT*_-*HYP3*, and all but one of them produced significantly more 1,8-cineole than the strain harboring the plant GPPS, P_*TEF1*_-*tAgGPPS2* (Fig. 4b). Mutants of the *S. cerevisiae* FPP synthase (ERG20) proved to be more effective than the corresponding mutants of the native *R. toruloides* ERG20, while the most promising enzyme of those tested was GgFPS(N144W) from *Gallus gallus* (chicken). The best strain harboring P_*ANT*_-*HYP3* and P_*TEF1*_-*GgFPS(N144W)*, 345, reached a 1,8-cineole titer of 143 mg/L.

Additional strains were constructed to test further configurations of *GgFPS(N144W)* and *HYP3* (Fig. 4c). Translational fusions of the two genes were expressed under control of P_*GAPDH*_ and although one configuration (strain 338, with GgFPS(N144W) N-terminal) was significantly better than the other (strain 340), the best 1,8-cineole titer was only 30% of that reached by strain 345. When P_*TEF1*_-*GgFPS(N144W)* was combined with *HYP3* under control of P_*GAPDH*_ rather than P_*ANT*_ (strain 343) the 1,8-cineole titer was surprisingly much lower, compared to that of strain 345. The combination of both P_*GAPDH*_-*HYP3* and P_*ANT*_-*HYP3* with P_*TEF1*_-*GgFPS(N144W)* via gene stacking (strain 342) resulted in a 1,8-cineole titer matching that of 345, indicating that perhaps a plateau had been reached that might only be surpassed by increasing flux through the mevalonate pathway.

### Targeted mevalonate pathway metabolic analysis

**Fig. 5:**
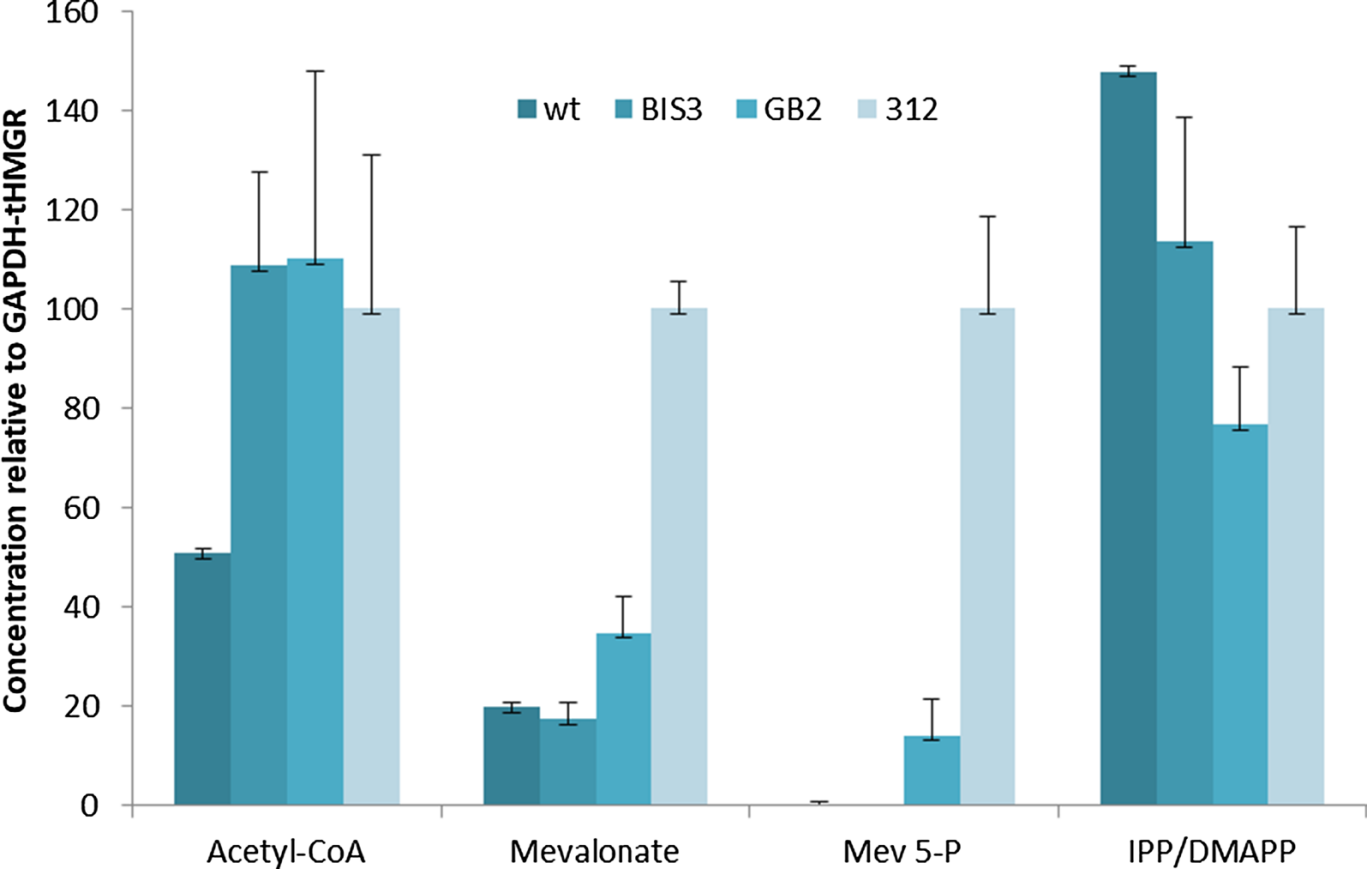
Mevalonate pathway intermediates in *R. toruloides* WT, BIS3, GB2, and 312 after 40 h of growth in SD medium containing 20 g/L glucose. Intracellular concentrations are expressed relative to strain 312; Mev 5-P, mevalonate 5-phosphate

Since this is a first attempt to engineer the mevalonate pathway in *R. toruloides*, we supplemented the global proteomic and transcriptomic data gathered from the α-bisabolene strains with targeted metabolomic analysis of mevalonate pathway intermediates to inform an engineering strategy. Little is known about mevalonate pathway regulation in this species and, particularly as omics data showed that several pathway genes (*ERG10*, *ERG13*, *ERG20*) were upregulated in response to α-bisabolene synthase overexpression (Fig. 2), it was considered prudent to avoid assumptions on rate limiting steps. At the same time, mevalonate pathway engineering strategies in *S. cerevisiae* and other fungi typically start with overexpression of 3-hydroxy-3-methyl-glutaryl-coenzyme A reductase (HMGR) and it was of interest to see how this would impact pathway metabolite levels [31–33]. Therefore, a new strain (312) to be included in the metabolomic analysis was constructed by the introduction of a truncated HMGR from *Cricetulus griseus* under control of the *GAPDH* promoter (P_*GAPDH*_-*tCgHMGR*) into strain GB2. Mevalonate pathway metabolites were analyzed for four strains, *R. toruloides* WT, BIS3, GB2, and 312, which were grown for 40 h to late exponential phase in SD medium containing 20 g/L glucose (Fig. 5). Intracellular levels of the early and late metabolites of the mevalonate pathway, acetyl-CoA and IPP/DMAPP, respectively, did not vary greatly between the α-bisabolene-producing strains. However, the acetyl-CoA concentration was higher in the α-bisabolene producing strains, compared to WT. Mevalonate accumulated in all strains, but was around 75% higher in strain GB2 and over fivefold higher in strain 312, compared to levels in *R. toruloides* WT. Mevalonate 5-phosphate did not accumulate in *R. toruloides* WT or BIS3 but was detected in GB2 and 312, with a sevenfold higher concentration in the latter strain. The approximate intracellular mevalonate concentration in strain 312 (27 µM) was around 20-fold higher than acetyl-CoA (1.6 µM) and mevalonate 5-phosphate (1.1 µM).

The data suggest that increasing production of α-bisabolene through high-level expression of *AgBIS* in strain GB2 promotes flux through the mevalonate pathway leading to an increase in mevalonate and mevalonate 5-phosphate levels. Since overexpression of *tCgHMGR* amplifies this effect, it seems likely that HMGR is somewhat rate limiting, as is the case in many other eukaryotes [31–33], but that production of mevalonate and mevalonate 5-phosphate emerge as downstream bottlenecks. Thus, HMGR and the two enzymes that process mevalonate and mevalonate 5-phosphate, MK and PMK, respectively, are possible overexpression targets for increasing mevalonate pathway flux in *R. toruloides*.

### Mevalonate pathway engineering

Orthologs of the central mevalonate genes (encoding HMGR, MK, or PMK) were selected for expression in *R. toruloides* based on their characteristics or success in engineering terpene production in other organisms. The promoters, P_*ANT*_, P_*SKP1*_ and P_*DUF*_ were selected for transgene expression based on previous characterization as very strong, strong, and medium strength (annotated as P15, P6, and P21, respectively, in that study) [15]. Prior to the combination of the candidate genes onto single constructs, they were tested individually for impact on α-bisabolene and 1,8-cineole titers by expression in strains GB2 and 345, respectively. Two HMGR candidates, from *Delftia acidovorans* (DaHMGR) and *Silicibacter pomeroyi* (SpHMGR), were the most impactful on 1,8-cineole titers when expressed in strain 345 under control of the two strongest promoters, P_*ANT*_ and P_*SKP1*_ (Additional file 1: Fig. S2a). SpHMGR was previously employed to engineer the mevalonate pathway in combination with central metabolism to increase terpene yields in *S. cerevisiae* [4] while DaHMGR was previously employed to engineer flux through a heterologous mevalonate pathway in *E. coli* [34]. Both are bacterial, NADH-dependent HMGRs that lack the membrane-bound domains of eukaryotic HMGRs. Expression of heterologous MK and PMK genes, however, had a less marked impact on terpene production; perhaps not too surprising as the metabolic analysis suggests that they might only become limiting when HMGR is overexpressed (Fig. 5). Nevertheless, orthologs were down selected for expression in combinatorial constructs based on the available data. MKs, responsible for conversion of mevalonate to mevalonate-5-phosphate, are often subject to feedback inhibition by prenyl phosphates or mevalonate diphosphate but several Archaeal MKs are not subject to this control mechanism [35]. An Archaeal MK, from *Methanosaeta concilii* (McMK), which was previously found to be kinetically (*k*_cat_ /K_m_) more favorable than MKs from other Archaea and *S. cerevisiae*, had the most favorable impact on α-bisabolene production in *R. toruloides* when under control of the medium-strong promoter, P_*SKP1*_ (Additional file 1: Fig. S2b) [35]. Thus, P_*ANT*_-*DaHMGR*, P_*ANT*_-*SpHMGR* and P_*SKP1*_-*McMK* were selected for expression and the remaining promoter, P_*DUF*_, was selected to drive expression of PMKs from *S. cerevisiae* (ScPMK) and *Streptococcus pneumoniae* (SpPMK) (Additional file 1: Fig. S2c) [36, 37].

**Fig. 6:**
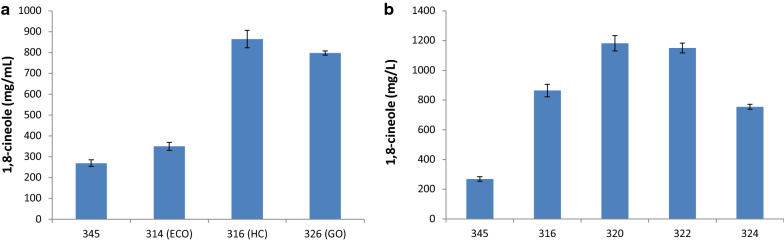
Boosting production of 1,8-cineole in *R. toruloides* by overexpression of central mevalonate pathway enzymes, HMGR, MK, and PMK. **a** Comparison of three different methods for codon optimization (ECO, HC, and GO, described in Materials and Methods) for constructs harboring P_*ANT*_-SpHMGR, P_*SKP1*_-McMK, and P_*DUF*_-ScPMK. **b** Various combinations of P_*ANT*_-HMGR, P_*SKP1*_-MK, and P_*DUF*_-PMK, described in Table 1, all codon-optimized using the HC method. Strains were cultivated in flower plates containing GXY medium and 1,8-cineole was measured at 7 days. HMGR, 3-hydroxy-3-methyl-glutaryl-coenzyme A reductase; MK, mevalonate kinase; PMK, phosphomevalonate kinase

Various combinations of the selected HMGR, MK and PMK expression cassettes were assembled onto single plasmid backbones for testing in the α-bisabolene and 1,8-cineole production strains, GB2 and 345, respectively. For one configuration of these genes (P_*ANT*_-SpHMGR, P_*SKP1*_-McMK, and P_*DUF*_-ScPMK), three versions were built using different methods for codon optimization of the three coding sequences. Transformation of these constructs into the 1,8-cineole producing strain, 345, revealed that an in-house codon optimization method (expression cassette optimization, ECO), based on a combination of learnings from literature and analysis of *R. toruloides* RNAseq and proteomics data, worked poorly while the approaches of using the most preferred codon in *R. toruloides* for each amino acid (high-CAI, HC) or Genscript’s codon optimization algorithm (Genscript-optimized, GO) produced similar results (Fig. 6a). The GO method is also strongly biased towards selection of the most preferred codons and the output DNA sequences for the HC and GO methods differed by only a few percent. The success of the high-CAI codon optimization methods is surprising, but has been borne out by other studies in *R. toruloides* [38]. Four constructs that contain HMGR, MK and PMK coding sequences, optimized using the HC method, were transformed into strain 345 by ATMT and the resulting strains were screened for 1,8-cineole production. Constructs pGEN-485 (P_*ANT*_-SpHMGR, P_*SKP1*_-McMK, P_*DUF*_-SpPMK) and pGEN-486 (P_*ANT*_-DaHMGR, P_*SKP1*_-McMK, P_*DUF*_-ScPMK) were the most successful of these, resulting in 1,8-cineole titers of over 1 g/L in strains 320 and 322 (Fig. 6b). The fact that similar titers were achieved with coupling of two different HMGR/PMK pairs suggests that pathway optimization may depend as much on achieving a balance between enzymes in the pathway as finding individual successful orthologs.

### Medium and process optimization

To test whether terpene production may be limited by the lack of certain nutrients in corn stover hydrolysate, strain GB2 was grown in DMR-EH media supplemented individually with each of the components present in yeast nitrogen base (Difco). Of the supplements, thiamine hydrochloride, pyridoxine hydrochloride, FeSO_4_, and Na_2_SO_4_ positively impacted production of α-bisabolene (Additional file 1: Fig. S3).

**Fig. 7:**
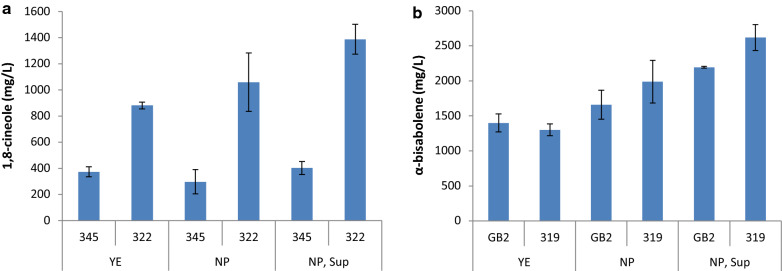
Optimization of DMR-EH based media for the production of terpenes in *R.* toruloides. Strains were cultivated in flower plates and 1,8-cineole (**a**) and α-bisabolene (**b**) were quantified at 6 days and 12 days, respectively. YE, 10 g/L yeast extract; NP, 5 g/L ammonium sulfate and 100 mM potassium phosphate (pH 6.0); Sup, supplementation with 400 µg/L thiamine hydrochloride, 400 µg/L pyridoxine hydrochloride, 100 µg/L FeSO_4_, and 1 mM Na_2_SO_4_. DMR-EH, Lignocellulosic hydrolysate prepared from corn stover by deacetylation and mechanical refining followed by enzymatic hydrolysis

Using microtiter plates with flower-shaped wells (flower plates) to enhance aeration, the highest titers were achieved in DMR-EH medium supplemented with 5 g/L (NH_4_)_2_SO_4_, 100 mM potassium phosphate (pH 6.0), 400 µg/L thiamine hydrochloride, 400 µg/L pyridoxine hydrochloride, 100 µg/L FeSO_4_, and 1 mM Na_2_SO_4_ (Fig. 7). Production of 1,8-cineole increased in both the 345 parent strain and the mevalonate-engineered strain 322, when cultured in the supplemented medium, with the latter producing 1.4 g/L. An α-bisabolene titer of 2.2 g/L was attained in strain GB2 and this improved to 2.6 g/L in the mevalonate-engineered strain 319.

### Reexamination of mevalonate pathway intermediates

Although the expression of HMGR, MK, and PMK orthologs in strain 345 yielded a fourfold increase in 1,8-cineole titer, transformation of the same constructs into GB2 had a more muted impact on the α-bisabolene titer. Targeted analysis of mevalonate pathway intermediates was employed to probe this disparity in impact on sesquiterpene versus monoterpene titers (Additional file 1: Fig. S4). Mevalonate, mevalonate 5-phosphate and mevalonate 5-diphosphate levels were all higher in the α-bisabolene producing parent strain, GB2, compared to the 1,8-cineole producing parent strain, 345. Upon overexpression of HMGR, MK, and PMK in these strains, intracellular levels of the same three pathway intermediates increased in both cases. However, higher levels were reached in the α-bisabolene producing strain, 319, compared to the 1,8-cineole producing strain, 317. As the increase in mevalonate pathway flux translates into a larger increase in monoterpene than sesquiterpene titers, it is possible that the discrepancy is related to the balance between the C_5_ precursors IPP and DMAPP. Production of the monoterpene precursor GPP requires a 1:1 ratio of IPP to DMAPP, while the sesquiterpene precursor FPP requires a 2:1 ratio, suggesting IPP/DMAPP isomerase (IDI) is a possible engineering target. Overexpression of IDI has proved effective in increasing titers of sesquiterpenes and carotenoids in other organisms, with data suggesting that it becomes more important as higher IPP/DMAPP ratios are required for production of larger terpenes [39, 40]. It is also possible that sesquiterpene production is limited by FPP synthase which, unlike GPP synthase in the 1,8-cineole producing strains, has not been overexpressed in the α-bisabolene strains. However, since the quantity of α-bisabolene produced in *R. toruloides* is twice that for 1,8-cineole, it is entirely possible that both terpenes are limited by a common factor, such as mevalonate diphosphate decarboxylase or by a nutrient in the DMR-EH medium. These will be among the factors to be evaluated in future engineering studies. A summary of key strains and metabolic pathway changes are shown in Additional file 1: Table S3 and Figure S5, respectively.

## Conclusions

The α-bisabolene and 1,8-cineole titers attained in *R. toruloides*, 2.6 and 1.4 g/L, respectively, appear to be the highest titers achieved for these terpenes in a microbial host to date. Importantly, they are achieved by growth on corn stover hydrolysate, supplemented only with 5 g/L ammonium sulfate and a few other nutrients. The DMR-EH medium contains approximately 115 g/L sugars (73 g/L glucose, 39 g/L xylose and 3 g/L arabinose) and the current yields for α-bisabolene and 1,8-cineole (0.022 and 0.012 g terpene/g sugars, or about 10% and 5% of the maximum biochemical yields via the mevalonate pathway, respectively) leave substantial room for improvement. A large amount of carbon is usually directed towards lipid synthesis in *R. toruloides* and then later recycled back to acetyl-CoA, as needed. This introduces a large degree of inefficiency to the terpene biosynthetic pathway, significantly reducing the maximum biochemical yield. Therefore, reduction of flux from acetyl-CoA to lipids remains a high priority for future work in this host, a feat that will require further development of genetic tools for downregulation of gene expression.

## Materials and methods

### Media

YPD medium (10 g/L yeast extract, 20 g/L peptone, and 20 g/L glucose), prepared using components from Difco (Becton, Dickinson and Company (BD), Franklin Lakes, NJ, USA), and lysogeny broth (LB, Miller formulation, VWR, Radnor, PA, USA) were used for routine growth of *R. toruloides*. YPD_10_ refers to YPD containing 100 g/L glucose. GXY medium contained 75 g/L glucose, 25 g/L xylose and 10 g/L yeast extract (Difco). Synthetic defined (SD) medium was prepared following manufacturers’ instructions, using Difco yeast nitrogen base (YNB) without amino acids (Becton, Dickinson & Co., Sparks, MD, USA) and complete supplement mixture (CSM; Sunrise Science Products, San Diego, CA, USA). Lignocellulosic hydrolysate was prepared from corn stover by deacetylation and mechanical refining followed by enzymatic hydrolysis, as described previously, and is referred to here as DMR-EH [13]. DMR-EH, supplied by the National Renewable Energy Laboratory (NREL), was centrifuged to remove solids and then sterilized by passage through a 0.2-µM filter before use. Strains were normally grown at 30 °C on solid medium containing 15 g/L agar, or in liquid medium with shaking at 200 rpm (Adolf Kühner AG, SBM-X, Birsfelden, Switzerland) unless strains were grown in plate format, in which case higher shaking speeds were employed (see *Terpene production and quantitation*, below).

### Strains, plasmids and transformation

*R. toruloides* IFO0880 and *Agrobacterium tumefaciens* EHA 105 were obtained from Jeffrey Skerker at UC Berkeley [14]. Binary plasmids for heterologous gene expression were built by GenScript (Piscataway, NJ, USA). Heterologous DNA was introduced into *R. toruloides* by *Agrobacterium tumefaciens*-mediated transformation (ATMT) as described previously [2] or in some cases by chemical transformation using lithium acetate [15]. In both cases, transformation results in integration of the heterologous DNA at random loci with variable copy numbers. Strains and plasmids used in this study can be found in Table 1 and are available upon request; more details, including plasmid maps, can be found on the Agile BioFoundry Strain Registry (http://public-registry.agilebiofoundry.org/ [16]).

**Table 1:** Overview of constructs used to engineer terpene production in *R. toruloides*

Genotype/features	Name	Registry ID
Plasmids		
P_*GAPDH*_-BIS-T_*NOS*_, NAT	GPD-BIS-NOS	JPUB_017422
P_*ANT*_-BIS-T_*NOS*_, HYG	pGEN-295	JPUB_017423
P_*ANT*_-HYP3-T_*NOS*_, HYG	pGEN-299	ABFPUB_000356
P_*TEF1*_-tAgGPPS2-T_*NOS*_, KAN	pGEN-376	ABFPUB_000335
P_*ANT*_-HYP3-T_*NOS*_, P_*TEF1*_-AgGPPS2-T_*35S*_, KAN	pGEN-308	ABFPUB_000348
P_*GAPDH*_-HYP3-T_*NOS*_, NAT	pGEN-300	ABFPUB_000357
P_*TEF1*_-AgGPPS2-T_*35S*_, BLE	pGEN-305	ABFPUB_000351
P_*ANT*_-HYP3-T_*NOS*_, P_*TEF1*_-tAgGPPS2-T_*35S*_, HYG	pGEN-309	ABFPUB_000353
P_*TEF1*_-RtERG20(F91W)-T_*NOS*_, KAN	pGEN-374	ABFPUB_000331
P_*TEF1*_-RtERG20(F91W, N122W)-T_*NOS*_, KAN	pGEN-375	ABFPUB_000333
P_*TEF1*_-ScERG20(F96W)-T_*NOS*_, KAN	pGEN-372	ABFPUB_000327
P_*TEF1*_-ScERG20(F96W, N127W)-T_*NOS*_, KAN	pGEN-373	ABFPUB_000329
P_*ANT*_-HYP3-T_*NOS*_, P_*TEF1*_-GgFPS(N144W)-T_*35S*_, NAT	pGEN-307	ABFPUB_000344
P_*GAPDH*_-HYP3-GgFPS(N144W)-T_*NOS*_ (fusion), NAT	pGEN-379	ABFPUB_000339
P_*GAPDH*_-GgFPS(N144W)-HYP3-T_*NOS*_ (fusion), NAT	pGEN-378	ABFPUB_000337
P_*TEF1*_-GgFPS(N144W)-T_*NOS*_, KAN	pGEN-304	ABFPUB_000341
P_*GAPDH*_-tCgHMGR-T_*NOS*_, KAN	GPD-tHMGR	ABFPUB_000355
P_*ANT*_-SpHMGR-T_*NOS*_, P_*SKP1*_-McMK-T_*SKP1*_, P_*DUF*_-ScPMK-T_*DUF*_ (ECO), KAN	pGEN-446	ABFPUB_000313
P_*ANT*_-SpHMGR-T_*NOS*_, P_*SKP1*_-McMK-T_*SKP1*_, P_*DUF*_-ScPMK-T_*DUF*_ (HC), KAN	pGEN-484	ABFPUB_000315
P_*ANT*_-SpHMGR-T_*NOS*_, P_*SKP1*_-McMK-T_*SKP1*_, P_*DUF*_-ScPMK-T_*DUF*_ (GO), KAN	pGEN-494	ABFPUB_000325
P_*ANT*_-SpHMGR-T_*NOS*_, P_*SKP1*_-McMK-T_*SKP1*_, P_*DUF*_-SpPMK-T_*DUF*_ (HC), KAN	pGEN-485	ABFPUB_000318
P_*ANT*_-DaHMGR-T_*NOS*_, P_*SKP1*_-McMK-T_*SKP1*_, P_*DUF*_-ScPMK-T_*DUF*_ (HC), KAN	pGEN-486	ABFPUB_000321
P_*ANT*_-DaHMGR-T_*NOS*_, P_*SKP1*_-McMK-T_*SKP1*_, P_*DUF*_-SpPMK-T_*DUF*_ (HC), KAN	pGEN-487	ABFPUB_000323
Strains		
* R. toruloides* IFO0880, mating type A2 (WT)		ABFPUB_000014
IFO0880/P_*GAPDH*_-BIS-T_*NOS*_ (1 copy)	BIS18	JPUB_013664
IFO0880/P_*GAPDH*_-BIS-T_*NOS*_ (7 copies)	BIS14	JPUB_017424
IFO0880/P_*GAPDH*_-BIS-T_*NOS*_ (10 copies)	BIS3	JPUB_009679
IFO0880/P_*GAPDH*_-BIS-T_*NOS*_, P_*ANT*_-BIS-T_*NOS*_	GB2	ABFPUB_000311
IFO0880/P_*ANT*_-HYP3-T_*NOS*_, P_*TEF1*_-tAgGPPS2-T_*35S*_	336	ABFPUB_000336
IFO0880/P_*ANT*_-HYP3-T_*NOS*__P_*TEF1*_-tAgGPPS2-T_*35S*_	350	ABFPUB_000350
IFO0880/P_*GAPDH*_-HYP3-T_*NOS*_, P_*TEF1*_-tAgGPPS2-T_*35S*_	352	ABFPUB_000352
IFO0880/P_*GAPDH*_-HYP3-T_*NOS*_, P_*TEF1*_-tAgGPPS2-T_*35S*__P_*ANT*_-HYP3-T_*NOS*_	354	ABFPUB_000354
IFO0880/P_*GAPDH*_-HYP3-T_*NOS*_, P_*TEF1*_-AgGPPS2-T_*35S*__P_*ANT*_-HYP3-T_*NOS*_	349	ABFPUB_000349
IFO0880/P_*ANT*_-HYP3-T_*NOS*_, P_*TEF1*_-RtERG20(F91W)-T_*NOS*_	332	ABFPUB_000332
IFO0880/P_*ANT*_-HYP3-T_*NOS*_, P_*TEF1*_-RtERG20(F91W, N122W)-T_*NOS*_	334	ABFPUB_000334
IFO0880/P_*ANT*_-HYP3-T_*NOS*_, P_*TEF1*_-ScERG20(F96W)-T_*NOS*_	328	ABFPUB_000328
IFO0880/P_*ANT*_-HYP3-T_*NOS*_, P_*TEF1*_-ScERG20(F96W, N127W)-T_*NOS*_	330	ABFPUB_000330
IFO0880/P_*ANT*_-HYP3-T_*NOS*__P_*TEF1*_-GgFPS(N144W)-T_*35S*_	345	ABFPUB_000345
IFO0880/P_*GAPDH*_-HYP3-GgFPS(N144W)-T_*NOS*_ (fusion)	340	ABFPUB_000340
IFO0880/P_*GAPDH*_-GgFPS(N144W)-HYP3-T_*NOS*_ (fusion)	338	ABFPUB_000338
IFO0880/P_*GAPDH*_-HYP3-T_*NOS*_, P_*TEF1*_-GgFPS(N144W)-T_*NOS*_	343	ABFPUB_000343
IFO0880/P_*ANT*_-HYP3-T_*NOS*_, P_*GAPDH*_-HYP3-T_*NOS*_, P_*TEF1*_-GgFPS(N144W)-T_*NOS*_	342	ABFPUB_000342
IFO0880/P_*GAPDH*_-BIS-T_*NOS*_, P_*ANT*_-BIS-T_*NOS*_, P_*GAPDH*_-tCgHMGR-T_*NOS*_	312	ABFPUB_000312
345/P_*ANT*_-SpHMGR-T_*NOS*__P_*SKP1*_-McMK-T_*SKP1*__P_*DUF*_-ScPMK-T_*DUF*_ (ECO)	314	ABFPUB_000314
345/P_*ANT*_-SpHMGR-T_*NOS*__P_*SKP1*_-McMK-T_*SKP1*__P_*DUF*_-ScPMK-T_*DUF*_ (HC)	316	ABFPUB_000316
345/P_*ANT*_-SpHMGR-T_*NOS*__P_*SKP1*_-McMK-T_*SKP1*__P_*DUF*_-ScPMK-T_*DUF*_ (GO)	326	ABFPUB_000326
345/P_*ANT*_-SpHMGR-T_*NOS*__P_*SKP1*_-McMK-T_*SKP1*__P_*DUF*_-SpPMK-T_*DUF*_ (HC)	320	ABFPUB_000320
345/P_*ANT*_-DaHMGR-T_*NOS*__P_*SKP1*_-McMK-T_*SKP1*__P_*DUF*_-ScPMK-T_*DUF*_ (HC)	322	ABFPUB_000322
345/P_*ANT*_-DaHMGR-T_*NOS*__P_*SKP1*_-McMK-T_*SKP1*__P_*DUF*_-SpPMK-T_*DUF*_ (HC)	324	ABFPUB_000324
345/P_*ANT*_-SpHMGR-T_*NOS*__P_*SKP1*_-McMK-T_*SKP1*__P_*DUF*_-ScPMK-T_*DUF*_ (HC)	317	ABFPUB_000317
GB2/P_*ANT*_-SpHMGR-T_*NOS*__P_*SKP1*_-McMK-T_*SKP1*__P_*DUF*_-SpPMK-T_*DUF*_ (HC)	319	ABFPUB_000319

Strains and plasmids used in this study are available upon request through strain registries of the Agile BioFoundry (http://public-registry.agilebiofoundry.org/) and the Joint BioEnergy Institute (https://public-registry.jbei.org/), designated by ABF and JBx, respectively [16]. Unless indicated, sequences are from *R. toruloides*, with the exception of antibiotic resistance genes. *GAPDH*, glyceraldehyde 3-phosphate dehydrogenase; BIS, α-bisabolene synthase from *Abies grandis* (NCBI Accession Number, O81086), *NOS*, nopaline synthase from *A. tumefaciens* (MK078637); *ANT*, adenine nucleotide translocase; *TEF1*, translational elongation factor; NAT, nourseothricin resistance cassette; HYG, Hygromycin B resistance cassette; KAN, kanamycin (G418) resistance cassette; *HYP3*, 1,8-cineole synthase from *Hypoxylon sp. E7406B* (AHY23922); AgGPPS2, GPP synthase from *A. grandis* (AAN01134); tAgGPPS2, AgGPPS2 with the N-terminal plastid transit peptide (84 amino acids) removed; 35S, 35S mRNA gene from cauliflower mosaic virus (KJ716236); RtERG20, FPP synthase from *R. toruloides* (PRQ75922); ScERG20, FPP synthase from *S. cerevisiae* (NP_012368); Gg*FPS*, FPP synthase from *Gallus gallus* (P08836.2); tCgHMGR, HMGR from *Cricetulus griseus*, truncated by removal of 351 N-terminal amino acids (XP_027257481), SpHMGR, HMGR from *Silicibacter pomeroyi* (WP_011241944); SKP1, S-phase kinase-associated protein 1 from *R. toruloides* (PRQ77980); McMK, MK from *Methanosaeta concilii* (WP_013720012); DUF, domain of unknown function from *R. toruloides* (PRQ75822); ScPMK, PMK from *S. cerevisiae* (AJS65138); SpPMK, PMK from *Streptococcus pneumoniae* (WP_044791288); and DaHMGR, HMGR from *Delftia acidovorans* (WP_099752490). Codon optimization was performed by Genscript, using a *R. toruloides* codon table, except where indicated by HC (high-CAI method) and ECO (expression cassette optimization). In the strain genotype description, a comma indicates sequential insertion (stacking) while an underscore indicates that the cassettes are on the same construct

### Codon optimization

Genes encoding terpene synthases and prenyl transferases were codon-optimized for *R. toruloides* by GenScript, based on a custom *R. toruloides* IFO0880 codon usage table (https://mycocosm.jgi.doe.gov/Rhoto_IFO0880_4/Rhoto_IFO0880_4.home.html). Genes encoding central mevalonate pathway enzymes were codon-optimized by GenScript, as above, and also by two other methods. The first of these (high codon adaptation index (high-CAI); HC) used the same codon usage table and *Codon Optimizer* software (genomes.urv.es/OPTIMIZER/) to select the codon most-utilized by *R. toruloides* for each amino acid. The second method (Expression Cassette Optimization, ECO) employed a balanced approach in which the second codon was optimized to approximate an optimal translational start consensus; less frequently used codons were selected for amino acids 3–10 in order to reduce mRNA secondary structure around the start codon; codons 11 to 45 and the last 50 codons were high-CAI and the remaining codons were selected to match the average usage in *R. toruloides*, using the same codon table and *Codon Optimizer* software [17–19].

### Terpene production and quantitation

Typically, *R. toruloides* strains were initially inoculated from YPD agar plates into LB medium and grown overnight to mid-log phase at 30 °C with shaking at 200 rpm. Optical density (OD) was determined by measuring absorbance at 600 nm (OD_600_) using a standard cuvette (10 mm lightpath) in a SpectraMax Plus 384 Microplate Reader (Molecular Devices, San Jose, CA, USA). For measurement of terpene production, strains were subcultured from the LB preculture into 5 mL production medium (YPD, YPD_10_, GXY, or DMR-EH) in 20 mm diameter culture tubes at a starting OD_600_ of 0.1. A 20% (v/v) dodecane (Sigma-Aldrich, St. Louis, MO, USA) overlay (1 mL) was added to capture terpenes produced during growth at 30 °C with shaking at 200 rpm. In some cases the dodecane overlay was spiked with an internal standard to adjust for evaporation—for production of 1,8-cineole, the dodecane overlay contained 1,4-cineole (Sigma-Aldrich) as an internal standard, while pentadecane (Sigma-Aldrich) was used as an internal standard for cultures producing α-bisabolene.

In some cases, growth and terpene production was carried out in 48-well flower plates (M2P-48-B, m2p-labs, Islandia, NY, USA) sealed with an Aeraseal air-permeable seal (Excel Scientific, Victorville, CA, USA), essentially as described above but employing a 1-mL culture volume and incubation at 30 °C, 1,000 rpm, and 70% relative humidity on a Multitron II platform shaker (Infors HT, Annapolis Junction, MD, USA).

For determination of 1,8-cineole product titers, the dodecane overlay was sampled and diluted in ethyl acetate spiked with 40 µg/mL pentadecane (to normalize for injection volume and instrument response during quantitation) followed by analysis by gas chromatography–mass spectrometry (GC–MS). For quantitation of α-bisabolene, the dodecane overlay was diluted into dodecane containing 40 µg/mL hexadecane (Sigma-Aldrich). GC–MS analysis was performed on an Agilent GC–MS 6890–5973 system (Agilent Technologies, Inc., Santa Clara, CA, USA) equipped with an Agilent DB-5 ms capillary column (30 m length, 0.25 mm diameter, 0.25 μm film thicknesses).

The separation and detection method for 1,8-cineole was as follows: 1 µL of sample was injected into the GC inlet in splitless mode with the GC oven temperature held at 80 °C for 1 min, ramped to 150 °C at 20 °C/min, and then ramped to 300 °C at 40 °C/min. The inlet purge flow rate was set to 11 mL/min at 0 min. The mass spectrometer was timed to turn off during elution of the solvents, ethyl acetate (0 to 2.5 min) and dodecane (4 to 5.8 min). The mass spectrometer was set to selected ion mode (SIM), targeting ions representative of 1,8-cineole and the internal standards 1,4-cineole and pentadecane, with mass/charge (m/z) ratios of 70, 85, 93, 136, 139, and 154. 1,8-cineole concentrations were calculated using an authentic 1,8-cineole (Sigma-Aldrich) standard curve after normalization of peak areas to those of the pentadecane internal standard. Evaporation of the 1,8-cineole product during production was corrected by quantitation of the 1,4-cineole that was spiked into the dodecane overlay at the outset of cell culture. The separation method for α-bisabolene [20] was performed as described previously, using an α-bisabolene standard prepared in-house. α-Bisabolene was quantified after normalization of peak areas to those of the hexadecane internal standard. In cases where the aeration rate was high during growth (e.g., during cultivation in a bioreactor), evaporation was corrected by quantification of the pentadecane that was spiked into the dodecane overlay at the outset of cell culture.

### Genome sequencing and determination of transgene copy number

Copy number of the integrated α-bisabolene synthase gene was determined from whole-genome resequencing of transformants. Whole-genome resequencing was performed at the Joint Genome Institute (JGI) using Illumina paired-end sequencing method using MiSeq 2 × 150 bp (JGI Proposal ID 503,063 and 1889). The sequence data have been deposited with the NCBI BioProject database under the following accession numbers: PRJNA370932 (BIS1), PRJNA370806 (BIS2), PRJNA370807 (BIS3), PRJNA370808 (BIS4), PRJNA370809 (BIS5), PRJNA370810 (BIS6), PRJNA370811 (BIS7), PRJNA370812 (BIS8), PRJNA370813 (BIS9), PRJNA370814 (BIS10), PRJNA370815 (BIS11), PRJNA370816 (BIS12), PRJNA370817 (BIS13), PRJNA370818 (BIS14), PRJNA370819 (BIS15), PRJNA371224 (BIS16), PRJNA370821 (BIS17), PRJNA370822 (BIS18), PRJNA370823 (BIS19), PRJNA371225 (BIS20), and PRJNA441663 (GB2).

The sequenced reads were mapped using BWA-MEM [21] to the reference genome sequence of *R. toruloides* IFO0880 (https://mycocosm.jgi.doe.gov/Rhoto_IFO0880_4/Rhoto_IFO0880_4.home.html) augmented with the sequence of the transformation plasmid containing P_*GAPDH*_-BIS or P_*ANT*_-BIS. The mapped reads were then sorted using SAMtools [22] and duplicate reads were marked using Picard Toolkit (Broad Institute, http://broadinstitute.github.io/picard) to generate the BAM files. The copy number of P_*GAPDH*_-BIS or P_*ANT*_-BIS was determined using CNVnator [23]. Bin sizes of 100, 200, and 1,000 bp were used for counting mapped reads within each bin and partitioning the read depth signal into segments. The average and standard deviation of read depth signal was evaluated for bin sizes of 100 and 200 bp, and copy number genotype was determined for the P_*GAPDH*_-BIS or P_*ANT*_-BIS region using each bin size. Estimated copy numbers were similar for bin sizes of 100 and 200 bps, and the results using 200 bp bin were used in Fig. 1.

### Bioreactor-scale cultivation for α-bisabolene production

Bioreactor cultivation was performed at the Advanced Biofuels Product Demonstration Unit (ABPDU) in a 2-L bioreactor (BIOSTAT B, Sartorius AG, Goettingen, Germany) containing 900 mL 75% DMR-EH supplemented with 10 g/L yeast extract (Difco) or defined nitrogen, as described in Results. A 20% (200 mL) dodecane overlay (v/v), spiked with 200 mg/L pentadecane as an internal standard, was added to the bioreactor to capture α-bisabolene produced during fermentation. The BIOSTAT B® fermentation system (Sartorius AG) was employed in batch mode, using a jacketed 2-L borosilicate glass vessel (UniVessel®, Sartorius AG) equipped with two 6-blade Rushton impellers, a dissolved oxygen (DO) probe (VisiFerm DO225, Hamilton Bonaduz AG, Bonaduz, Switzerland), and a pH probe (EasyFerm Plus VP 225, Hamilton Bonaduz AG). The bioreactor was inoculated to an OD_600_ of 1 from a culture propagated to mid-log phase on the same medium in 250-mL shake flasks (approx. 100 mL). Fermentation was carried out at 30 °C with air supplied at a sparge rate of 0.5 lpm (0.5 vvm) and constant agitation at 400 rpm. After initial adjustment of the growth medium pH to 6.0, the pH was maintained at or above 5.0 using 2 N sodium hydroxide. Process values were monitored and recorded using the integrated Sartorius data acquisition software (BioPAT MFCS/win). Sugar consumption, OD, and α-bisabolene production were measured over a period of 12 days.

### Quantification of xylose and glucose

Prior to analysis, samples were diluted as appropriate and filtered through 0.45 μm filters (VWR Centrifugal Filters) by centrifugation at 3000×g for 3 min. Sugars were quantified on a Dionex Ultimate 3000 system UHPLC (Agilent Technologies) using an Aminex HPX-87H column (Bio-Rad, Hercules, CA, USA) and Thermo Scientific™ RefractoMax 520 Refractive Index Detector (RID) (Thermo Fisher Scientific, Waltham, MA, USA) as described previously [5].

### Transcriptomics and proteomics analysis

Strains were cultivated in SD medium containing 1% (10 g/L) glucose. Regular samples were taken for measurement of growth and α-bisabolene titer, and the samples taken at 18 and 48 h post-inoculation were selected for transcriptomics and proteomics analysis, representing the exponential and stationary phases of growth. For transcriptomics analysis, 1-mL samples were centrifuged and cells were washed three times with DEPC-treated water before flash freezing in liquid nitrogen. For proteomics analysis, cells were washed three times with 100 mM ammonium bicarbonate and flash frozen in liquid nitrogen until usage.

Total RNA was isolated by disrupting harvested cells using an MP Biomedicals FastPrep bead beater (MP Biomedicals, Irvine, CA, USA) followed by purification using the QIAGEN RNeasy Mini kit with on-column DNase treatment following the manufacturer’s protocol. 10 µg of RNA was then used to prepare a sequencing library with the TruSeq RNA Library Preparation Kit v2 (Illumina, San Diego, CA, USA). The quality and concentrations of RNA samples and sequencing libraries were measured with an Agilent Bioanalyzer 2100 and Qubit. In addition, a q-PCR quantification of sequencing libraries was run using a KAPA library quantification kit (Roche, Pleasanton, CA, USA). RNA-sequencing was performed at the Agile BioFoundry using Illumina paired-end sequencing method using MiSeq 2 × 75 bp. The sequence data have been deposited with the NCBI GEO database under accession number GSE156051 (Token for reviewers to access the data: krmpyckyhxqdfop). Sequenced RNA reads were trimmed and filtered using BBTools (https://jgi.doe.gov/data-and-tools/bbtools/), and mapped to the reference genome sequence of *R. toruloides* IFO0880 augmented with the coding sequence of BIS using HISAT2 [24], Mapped reads were assigned to genes using featureCounts [25], and read counts were used to calculate Fragments Per Kilobase of transcript per Million mapped reads (FPKM).

For global proteomics analysis, to extract the protein from the cell pellets for global proteomics analysis, 1 mL of a mixture of chloroform:methanol (prepared 2:1 (v/v)) was pipetted into chloroform compatible 2 mL Sorenson MulTI™ SafeSeal™ microcentrifuge tubes (Sorenson bioscience, Salt Lake City, UT, USA) kept inside an ice block. Cell pellets were resuspended in 200 µL of water and immediately transferred into the microcentrifuge tubes. Samples were vigorously vortexed, placed in an ice block for 10 min, vortexed again for 20 s and centrifuged at 17,000×*g* for 10 min at 4 °C. The upper and lower phases were removed. The remaining protein interphase was washed with methanol, vortexed and centrifuged at 17,000×*g* for 5 min to pellet the protein. The methanol supernatant was decanted into waste and the pellet lightly dried in a fume hood and then stored at − 80 °C until protein digestion. The protein interlayer pellet was digested by adding 200 µL of an 8 M urea solution to the protein pellets and vortexed into solution. A bicinchoninic acid (BCA) assay (Thermo Fisher Scientific) was performed to determine protein concentration. Following the assay, 10 mM dithiothreitol (DTT) was added to the samples and incubated at 60 °C for 30 min with constant shaking at 800 rpm. Reduced cysteine residues were alkylated by adding 400 mM iodoacetamide (Sigma-Aldrich) to a final concentration of 40 mM and incubating in the dark at room temperature for 1 h. Samples were then diluted eightfold for preparation for digestion with 100 mM NH_4_HCO_3_, 1 mM CaCl_2_ and sequencing-grade modified porcine trypsin (Promega, Madison, WI, USA) was added to all protein samples at a 1:50 (w/w) trypsin-to-protein ratio for 3 h at 37 °C. Digested samples were desalted using a 4-probe positive pressure Gilson GX-274 ASPEC™ system (Gilson Inc., Middleton, WI, USA) with Discovery C18 100 mg/1 mL solid phase extraction tubes (Supelco, St. Louis, MO, USA), using the following protocol: 3 mL of methanol was added for conditioning followed by 2 mL of 0.1% TFA in H_2_O. The samples were then loaded onto each column followed by 4 mL of 95:5: H_2_O:ACN, 0.1% TFA. Samples were eluted with 1 mL 80:20 ACN:H_2_O, 0.1% TFA. The samples were concentrated down to ~ 100 µL using a Speed Vac and a final BCA was performed to determine the peptide concentration, and then stored at − 80 °C until usage. Peptides digests were diluted to 0.1 µg/uL with nanopure water for LC–MS/MS analysis. 5 µL of samples were loaded onto in-house packed reversed-phase capillary columns (70-cm × 75 µm i.d.) with 3-µm Jupiter C18 particles. The separation was carried out using a nanoAcquity HPLC system (Waters Corporation, Milford, MA, USA) at room temperature. The mobile phase A was 0.1% formic acid in water while the mobile phase B was 0.1% formic acid in acetonitrile. The elution was carried out at 300 nL/min with the following gradient: 0–2 min 1% B; 2–20 min 8% B; 20–75 min 12% B; 75–97 min 30% B; 97–100 min 45% B; 100–105 min 95% B; 110–140 min 1% B. The eluting peptides were directly analyzed using a Q Exactive Plus mass spectrometer (Thermo Fisher Scientific) in data-dependent acquisition mode. Mass spectrometer settings were as following: full MS (AGC, 3 × 10^6^; resolution, 35,000; m/z range, 400–2,000; maximum ion time, 20 ms); MS/MS (AGC, 1 × 10^5^; resolution, 17,500; m/z range 200–2000; maximum ion time, 100 ms; TopN, 12; isolation width, 2 Da; dynamic exclusion, 30.0 s; collision energy, NCE 30). All mass spectrometry data were searched using MS-GF + and MASIC software. MS-GF + software was used to identify peptides by scoring MS/MS spectra against peptides derived from the whole protein sequence database. MASIC software was used to generate the selected ion chromatographs (SICs) of all the precursors in MSMS datasets and calculate their peak areas as abundance. MASIC Results Merger (https://omics.pnl.gov/software/masic-results-merger) was used to append the relevant MASIC stats for each peptide-hit result in MS-GF+. The MS-GF + data were then filtered based on 1% false discovery rate (FDR) and less than 5 ppm mass accuracy to generate a list of qualified peptide-hit results. The abundance of peptides was determined as the highest peak area identified for the peptide within a sample. Sample level quality was ensured by a Mahalanobis distance (rMd), which evaluates multiple statistical parameters associated with each peptide profile for each sample, as well as sample-level Pearson correlation and overall peptide intensity. Peptides were also filtered to remove those with inadequate data for statistics, where a peptide was required to have at least 2 observations in 2 of the groups in Table 1, or 3 observations within a single group. Protein quantification was performed using a standard reference-based approach. For time comparison within strains, we utilized two-sample t-tests with a Bonferroni correction. For strain comparison at the same time point, a one-way ANOVA with a Dunnett test was performed.

### Targeted mevalonate pathway metabolite analysis

Strains *R. toruloides* WT, GB, GB2, and 312 were inoculated from LB precultures into SD media containing 20 g/L glucose and a 20% (v/v) dodecane overlay. At 40 h, ODs were measured and a 6 OD_600_ cell pellet was collected from each culture by centrifugation in a microfuge at 5000 rpm for 1 min at room temperature and then cells were quenched by adding 300 µL of cold methanol. Samples were vortexed for 10 s and then 300 µL chloroform was added, followed by another 10 s of vortexing. 150 µL water was added, followed by another mix by vortexing for 10 s. All solvents were liquid chromatography grade purity. Samples were centrifuged for 10 min at 14,000 rpm at 4 °C and the top aqueous layer was removed for metabolomics analysis. Water was added to bring the methanol/water ratio to 1:1 and samples were spun through Amicon 3000 molecular weight cut-off filters (Millipore Sigma, Burlington, MA, USA) and then lyophilized overnight and resuspended in 50 µL of a sample solvent composed of 60% acetonitrile, 30% water, and 10% methanol. Samples were analyzed alongside mevalonate pathway standards using a hydrophilic interaction liquid chromatography time-of-flight mass spectrometry (HILIC-TOF–MS) method described previously [26].

## Supplementary Information

**Additional file 1: **Supplemental Figures and Tables.

## Data Availability

All data generated or analyzed during this study are included in this published article and its supplementary information files.
