# Supplementary material for: Further engineering of R. toruloides for the production of terpenes from lignocellulosic biomass
Source: Biotechnol Biofuels. 2021 Apr 21;14:101. doi: 10.1186/s13068-021-01950-w (PMC8058980; doi:10.1186/s13068-021-01950-w)
Supplement: Supplementary file 1 — Additional file 1: Supplemental Figures and Tables. [file 13068_2021_1950_MOESM1_ESM.docx]

**Further engineering of *R. toruloides* for the production of terpenes from lignocellulosic biomass**

James Kirby^a,b^, Gina M. Geiselman^a,b^, Junko Yaegashi^c,d^, Joonhoon Kim^a,d^, Xun Zhuang^a,b^, Mary Bao Tran-Gymfi^a,b^, Jan-Philip Prahl^a,e^, Eric R. Sundstrom^a,e^, Yuqian Gao^a,i^, Nathalie Munoz^a,h^, Kristin E. Burnum-Johnson^a,h^, Veronica T. Benites^a,f^, Edward E. K. Baidoo^a,f^, Anna Fuhrmann^c^, Katharina Seibel^c^, Bobbie-Jo M. Webb-Robertson^a,i^, Jeremy Zucker^a,i^, Carrie D. Nicora^a,i^, Deepti Tanjore^a,e^, Jon K. Magnuson^a,d^, Jeffrey M. Skerker^g^ and John M. Gladden^a,b *^

**Affiliations**

^a^Department of Energy, Agile BioFoundry, Emeryville, CA, 94608, USA

^b^Department of Biomass Science and Conversion Technology, Sandia National Laboratories, Livermore, CA 94550, USA

^c^Joint BioEnergy Institute, Lawrence Berkeley National Laboratory, Emeryville, CA 94608, USA

^d^Chemical and Biological Processing Group, Pacific Northwest National Laboratory, Richland, WA 99354, USA

^e^Advanced Biofuels and Bioproducts Process Development Unit, Lawrence Berkeley National Laboratory, Emeryville, CA 94608, USA

^f^Biological Systems and Engineering Division, Lawrence Berkeley National Laboratory, Berkeley, CA 94720, USA

^g^Environmental Genomics and Systems Biology Division, Lawrence Berkeley National Laboratory, Berkeley, CA 94720, USA

^g^QB3-Berkeley, University of California, Berkeley, CA 94704, USA

^h^The Environmental Molecular Sciences Laboratory, ^i^Biological Sciences Division, Pacific Northwest National Laboratory, Richland, WA 99354, USA

^*^Corresponding author. Tel. +1 (510) 495-2490

Email address: (John M. Gladden) [jmgladden@lbl.gov](mailto:jmgladden@lbl.gov)


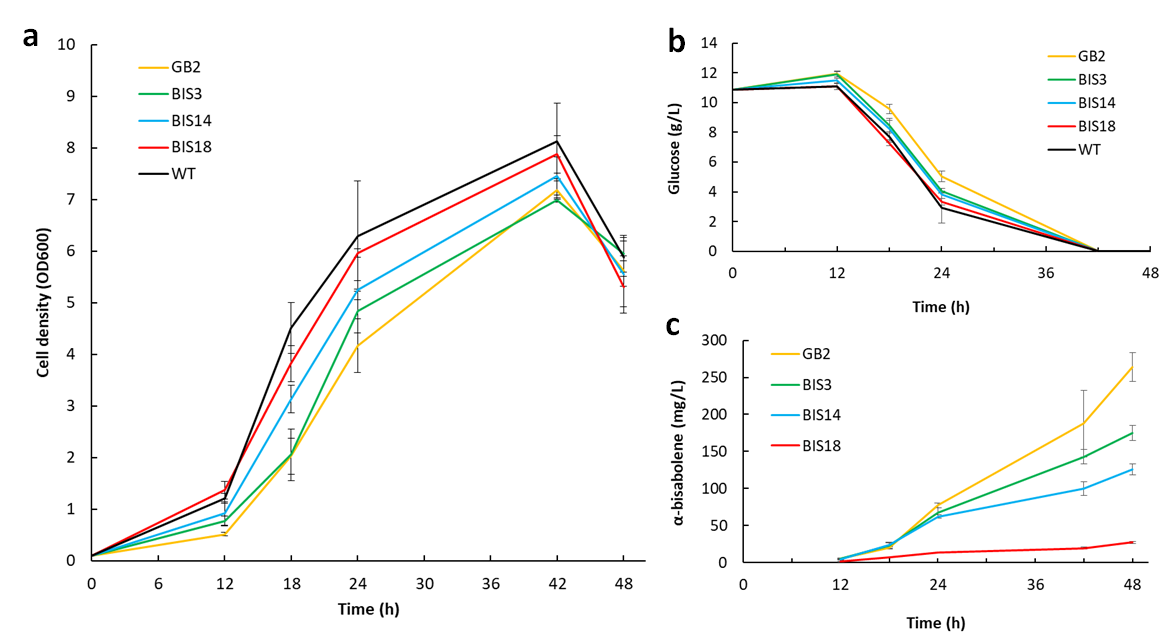


**Fig. S1** Growth (**a**), glucose consumption (**b**), and α-bisabolene production (**c**) in SD medium containing 10 g/L glucose by five *R. toruloides* strains selected for omics analysis, including wild type (WT). Three strains (BIS18, BIS14, and BIS3) harbor the P*_GAPDH_*-BIS cassette at various copy numbers (1, 7, and 10, respectively) and strain GB2 contains of 6 copies of P*_ANT_*-BIS in addition to 10 copies of P*_GAPDH_*-BIS.

**Table S1** Selected transcriptomics (A) and proteomics (B) data for five *R. toruloides* strains (WT, BIS18, BIS14, BIS3, and GB2) sampled following 18 hours (exponential, exp) and 48 hours (stationary, stat) of growth in SD medium containing 10 g/L glucose. The average of log2(FPKM) values and spectral counts for three replicates are used for transcriptomics and proteomics, respectively.

| A. | WT | BIS18 | BIS14 | BIS3 | GB2 | WT | BIS18 | BIS14 | BIS3 | GB2 |
| --- | --- | --- | --- | --- | --- | --- | --- | --- | --- | --- |
|  | exp | exp | exp | exp | exp | stat | stat | stat | stat | stat |
| ANT | 11.3 | 11.0 | 11.4 | 12.0 | 11.9 | 9.5 | 10.5 | 10.7 | 10.4 | 10.4 |
| BIS |  | 8.5 | 11.7 | 12.8 | 13.3 |  | 8.3 | 11.3 | 11.8 | 12.6 |
| TEF1 | 11.1 | 10.9 | 11.4 | 11.9 | 12.1 | 10.2 | 10.7 | 11.0 | 10.6 | 10.9 |
| ERG10 | 6.9 | 7.0 | 7.8 | 8.3 | 8.9 | 5.6 | 5.8 | 6.3 | 5.9 | 6.5 |
| ERG13 | 6.1 | 6.2 | 7.7 | 8.7 | 9.6 | 5.5 | 5.7 | 6.0 | 6.2 | 6.4 |
| ERG20 | 6.1 | 6.2 | 7.2 | 8.0 | 8.3 | 4.9 | 5.4 | 5.5 | 6.1 | 5.8 |

| B. | WT | BIS18 | BIS14 | BIS3 | GB2 | WT | BIS18 | BIS14 | BIS3 | GB2 |
| --- | --- | --- | --- | --- | --- | --- | --- | --- | --- | --- |
|  | exp | exp | exp | exp | exp | stat | stat | stat | stat | stat |
| ANT | 73.3 | 73.5 | 68.0 | 71.8 | 68.0 | 62.0 | 59.3 | 58.8 | 56.5 | 53.3 |
| BIS |  | 101.8 | 228.8 | 265.3 | 337.3 |  | 92.5 | 194.0 | 258.3 | 296.0 |
| TEF1 | 181.3 | 178.5 | 174.5 | 189.3 | 210.0 | 130.0 | 134.5 | 121.3 | 132.3 | 145.0 |
| ERG10 | 26.8 | 23.5 | 25.0 | 32.8 | 42.5 | 21.5 | 25.3 | 26.3 | 28.8 | 32.0 |
| ERG13 | 31.5 | 27.3 | 31.5 | 50.3 | 56.8 | 19.0 | 18.5 | 21.5 | 25.0 | 30.8 |
| ERG20 | 14.0 | 13.0 | 15.0 | 19.8 | 19.8 | 8.8 | 9.5 | 12.0 | 14.8 | 12.5 |

**Table S2** Additional transcriptomics and proteomics data for five *R. toruloides* strains (WT, BIS18, BIS14, BIS3, and GB2) sampled following 18 hours of growth in SD medium containing 10 g/L glucose. Transcript and protein data is shown as log2 fold change values for each of the engineered strains, compared to WT. Protein IDs correspond to entries in the reference genome sequence of *R. toruloides* IFO0880 (<https://mycocosm.jgi.doe.gov/Rhoto_IFO0880_4/Rhoto_IFO0880_4.home.html>)

|  |  | Transcriptomics | | | | | | | | Proteomics | | | | | | | |
| --- | --- | --- | --- | --- | --- | --- | --- | --- | --- | --- | --- | --- | --- | --- | --- | --- | --- |
|  |  | Log2 Fold Change (vs WT) | | | | Adjusted p-value | | | | Log2 Fold Change (vs WT) | | | | Adjusted p-value | | | |
| Protein ID | Name | **BIS18** | **BIS14** | **BIS3** | **GB2** | **BIS18** | **BIS14** | **BIS3** | **GB2** | **BIS18** | **BIS14** | **BIS3** | **GB2** | **BIS18** | **BIS14** | **BIS3** | **GB2** |
| 9726 | ACLY | -0.01 | 0.80 | 0.81 | 1.58 | 9.95E-01 | 1.66E-13 | 2.79E-14 | 2.04E-56 | 0.21 | 0.21 | 0.58 | 0.98 | 2.76E-02 | 2.73E-02 | 4.21E-06 | 2.48E-06 |
| 8639 | ACC1 | -0.22 | 0.54 | 0.65 | 1.24 | 6.88E-01 | 1.46E-03 | 5.01E-05 | 9.37E-18 | 0.17 | 0.22 | 0.24 | 0.60 | 3.17E-01 | 1.62E-01 | 1.09E-01 | 1.40E-04 |
| 8670 | FAS1 | -0.11 | 0.38 | 0.48 | 0.86 | 8.70E-01 | 2.38E-02 | 2.07E-03 | 6.86E-10 | 0.07 | 0.26 | 0.21 | 0.40 | 8.62E-01 | 5.69E-02 | 1.53E-01 | 3.66E-03 |
| 8777 | FAS2 | -0.22 | 0.79 | 0.81 | 1.80 | 7.67E-01 | 5.16E-05 | 1.31E-05 | 2.10E-26 | 0.16 | 0.35 | 0.28 | 0.79 | 3.87E-01 | 1.55E-02 | 6.28E-02 | 9.20E-06 |
| 8678 | ERG10 | 0.07 | 0.93 | 1.49 | 1.98 | 9.33E-01 | 2.06E-09 | 4.11E-25 | 2.63E-46 | 0.07 | 0.45 | 0.61 | 0.95 | 9.77E-01 | 5.27E-02 | 8.09E-03 | 1.65E-04 |
| 12122 | ERG13 | 0.05 | 1.60 | 2.67 | 3.46 | 9.70E-01 | 6.55E-17 | 3.42E-49 | 2.05E-84 | -0.21 | -0.07 | 1.00 | 1.12 | 5.61E-01 | 9.84E-01 | 1.33E-04 | 4.40E-05 |
| 9574 | HMGR | -0.18 | 0.41 | 0.74 | 1.29 | 7.67E-01 | 1.50E-02 | 2.86E-07 | 1.53E-24 | 0.14 | 0.12 | 0.22 | 0.89 | 9.32E-01 | 9.67E-01 | 7.65E-01 | 7.29E-03 |
| 8758 | MK | -0.06 | 0.51 | 0.34 | -0.76 | 9.41E-01 | 4.39E-03 | 6.56E-02 | 3.90E-06 | 0.14 | 0.11 | 0.19 | -0.36 | 6.65E-01 | 8.02E-01 | 4.24E-01 | 4.73E-02 |
| 8702 | PMK | 0.05 | -0.33 | -0.32 | 0.16 | 9.40E-01 | 3.49E-02 | 3.25E-02 | 2.49E-01 | -0.25 | 0.00 | 0.40 | -0.22 | 9.05E-01 | 1.00E+00 | 6.51E-01 | 9.36E-01 |
| 11910 | MVD | -0.20 | 0.28 | 0.34 | 1.00 | 8.03E-01 | 2.55E-01 | 1.19E-01 | 4.50E-09 | 0.32 | 0.03 | 0.86 | 0.05 | 3.50E-01 | 1.00E+00 | 2.49E-03 | 9.96E-01 |
| 16203 | IDI | 0.26 | 0.73 | 0.69 | 0.67 | 6.43E-01 | 3.81E-05 | 6.07E-05 | 3.51E-05 | 0.23 | 0.32 | 0.56 | 1.07 | 8.15E-01 | 6.01E-01 | 1.68E-01 | 4.49E-03 |
| 12944 | ERG20 | 0.14 | 1.08 | 1.93 | 2.26 | 8.30E-01 | 6.21E-13 | 2.74E-45 | 1.13E-64 | 0.25 | 0.33 | 0.85 | 0.85 | 3.18E-01 | 1.28E-01 | 1.63E-04 | 1.60E-04 |
| 16591 | ERG9 | -0.04 | 0.15 | 0.43 | 0.82 | 9.62E-01 | 4.17E-01 | 2.24E-03 | 1.68E-12 | -0.19 | -0.22 | -0.01 | -0.13 | 8.01E-01 | 7.19E-01 | 1.00E+00 | 9.42E-01 |
| 16503 | GGPPS | 0.22 | 0.49 | 0.47 | 0.57 | 6.27E-01 | 1.30E-03 | 1.58E-03 | 1.86E-05 | -0.22 | 0.08 | 0.00 | 0.20 | 5.53E-01 | 9.74E-01 | 1.00E+00 | 6.22E-01 |
| 13729 | ERG1 | 0.15 | 0.86 | 1.26 | 1.54 | 8.11E-01 | 1.72E-08 | 8.32E-19 | 7.52E-30 | 0.04 | 0.19 | 1.11 | 1.14 | 1.00E+00 | 9.43E-01 | 1.70E-02 | 1.46E-02 |
| 9240 | ERG7 | 0.57 | 1.42 | 2.46 | 2.44 | 1.13E-01 | 3.10E-14 | 1.43E-45 | 3.74E-46 | 0.45 | 0.21 | 1.03 | 1.11 | 1.32E-01 | 6.90E-01 | 4.93E-04 | 2.34E-04 |
| 12843 | ERG11 | 0.04 | 0.47 | 0.99 | 1.39 | 9.59E-01 | 5.97E-03 | 1.82E-11 | 7.69E-24 | -0.21 | 0.16 | 0.63 | 0.64 | 8.77E-01 | 9.46E-01 | 1.41E-01 | 1.30E-01 |


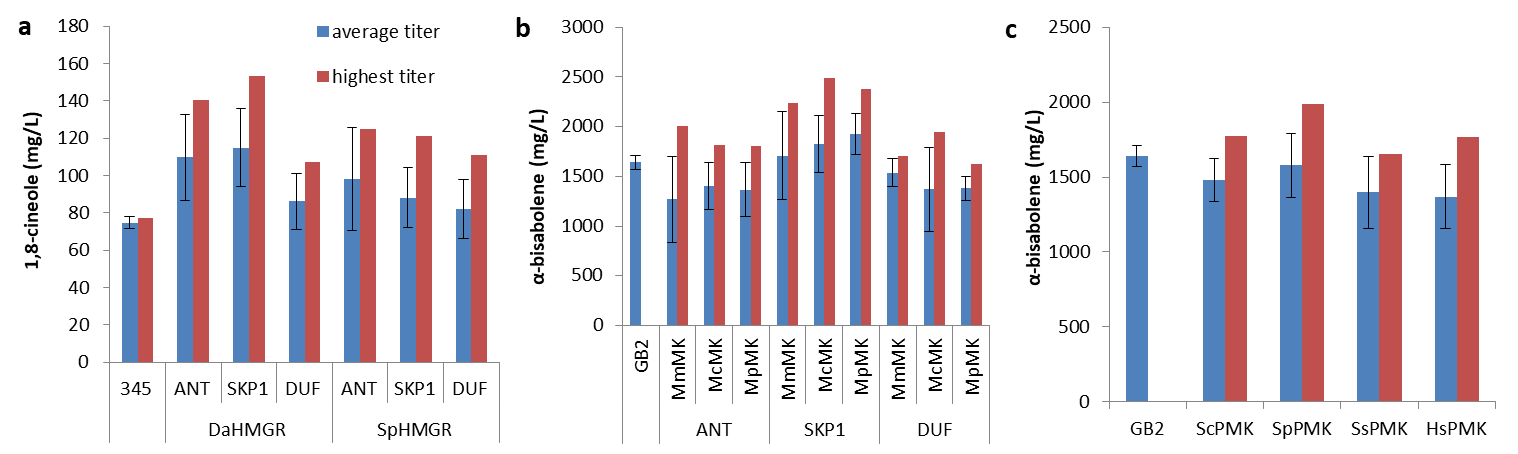


**Fig. S2** Determining the impact of HMGR, MK, and PMK ortholog overexpression on α-bisabolene and 1,8-cineole production in *R. toruloides* strains, GB2 and 345, respectively. **a** Native promoters P*_ANT_*, P*_SKP1_* and P*_DUF_* were used to drive expression of HMGRs from *Delftia acidovorans* (DaHMGR) and *Silicibacter pomeroyi* (SpHMGR) in the 1,8-cineole producing strain 345. **b** Archaeal MKs, from *Methanosarcina mazei* (MmMK), *Methanosaeta concilii* (McMK), and *Methanocella paludicola* (MpMK) were expressed under control of the P*_ANT_*, P*_SKP1_* and P*_DUF_* promoters in the α-bisabolene producing strain, GB2. **c** PMKs selected from *S. cerevisiae* (ScPMK), *Streptococcus pneumoniae* (SpPMK), *Sus scrofa* (SsPMK), and *Homo sapiens* (HsPMK) were expressed under control of P*_DUF_* in strain GB2. Strains were grown in culture tubes containing GXY medium and 1,8-cineole and α-bisabolene were measured at 8 and 7 days, respectively. Product titers are shown for the two parent strains, 345 and GB2, and as an average of all transformants for each gene inserted into these parents. The highest product titer reached by a single transformant is also shown for each of the HMGR, MK, and PMK orthologs expressed here.


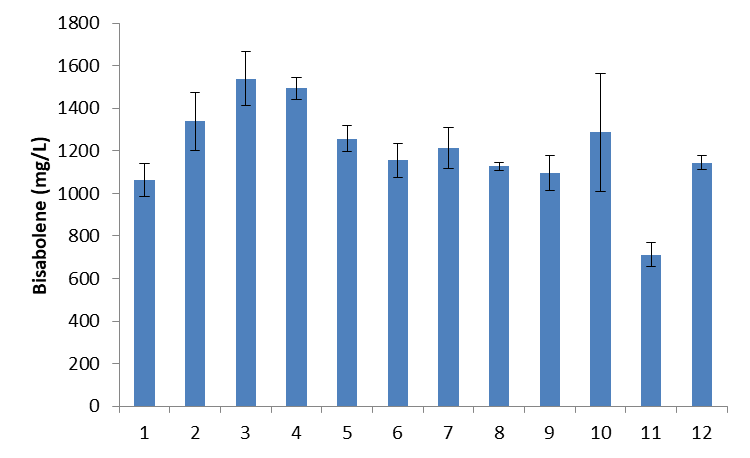


**Fig. S3** Production of α-bisabolene by strain GB2 in media composed of DMR-EH, 5 g/L (NH_4_)_2_SO_4_, 76 mM KH_2_PO_4_, 24 mM K_2_HPO_4_, pH 5.75_,_ supplemented with: nothing (1), 200 µg/L thiamine (2), 400 µg/L thiamine (3), 800 µg/L thiamine (4), 400 µg/L pyridoxine hydrochloride (5), 2 µg/L folic acid (6), 100 µM FeSO_4_ (7), 100 µM MgSO_4_ (8), 1 mM MgSO_4_ (9), 1 mM Na_2_SO_4_ (10), 7 mg/L ZnSO_4_ (11), or 6.2 mg/L CuSO_4_ (12).


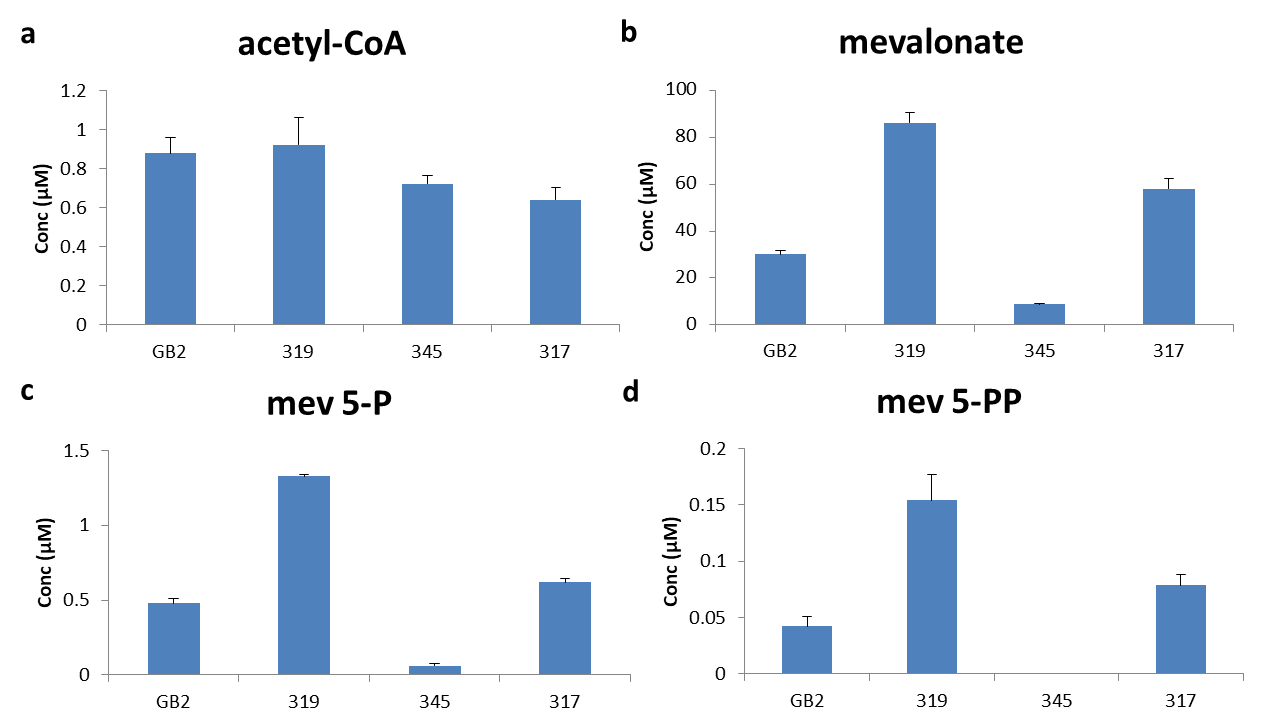


**Fig. S4** Accumulation of mevalonate pathway intermediates in response to mevalonate pathway engineering. Intracellular mevalonate pathway intermediates were measured for *R. toruloides* strains GB2, 319, 345, and 317 after 40 hours of growth in DMR-EH medium supplemented with 10 g/L yeast extract. Mev 5-P, mevalonate 5-phosphate; Mev 5-PP, mevalonate 5-diphosphate.

**Table S3** Key strains developed during this work. This is a subset of the strains listed in Table 1.

| **Genotype/features** | **Name** | **Registry ID** |
| --- | --- | --- |
| *R. toruloides* IFO0880, mating type A2 (wild type parent of all strains) | WT | ABFPUB_000014 |
|  |  |  |
| **Key *R. toruloides* strains engineered to produce α-bisabolene** |  |  |
| IFO0880/P*_GAPDH_*-BIS-T*_NOS_* (10 copies) | BIS3 | JPUB_009679 |
| IFO0880/P*_GAPDH_*-BIS-T*_NOS_*, P*_ANT_*-BIS-T*_NOS_* | GB2 | ABFPUB_000311 |
| GB2/P*_ANT_*-SpHMGR-T*_NOS_*_P*_SKP1_*-McMK-T*_SKP1_*_P*_DUF_*-SpPMK-T*_DUF_* (HC) | 319 | ABFPUB_000319 |
|  |  |  |
| **Key *R. toruloides* strains engineered to produce 1,8-cineole** |  |  |
| IFO0880/P*_ANT_*-HYP3-T*_NOS_*, P*_GAPDH_*-HYP3-T*_NOS_*, P*_TEF1_*-GgFPS(N144W)-T*_NOS_* | 342 | ABFPUB_000342 |
| IFO0880/P*_ANT_*-HYP3-T*_NOS_*_P*_TEF1_*-GgFPS(N144W)-T*_35S_* | 345 | ABFPUB_000345 |
| 345/P*_ANT_*-DaHMGR-T*_NOS_*_P*_SKP1_*-McMK-T*_SKP1_*_P*_DUF_*-SpPMK-T*_DUF_* (HC) | 324 | ABFPUB_000324 |
| 345/P*_ANT_*-SpHMGR-T*_NOS_*_P*_SKP1_*-McMK-T*_SKP1_*_P*_DUF_*-ScPMK-T*_DUF_* (HC) | 316 | ABFPUB_000316 |
| 345/P*_ANT_*-DaHMGR-T*_NOS_*_P*_SKP1_*-McMK-T*_SKP1_*_P*_DUF_*-ScPMK-T*_DUF_* (HC) | 322 | ABFPUB_000322 |
| 345/P*_ANT_*-SpHMGR-T*_NOS_*_P*_SKP1_*-McMK-T*_SKP1_*_P*_DUF_*-SpPMK-T*_DUF_* (HC) | 320 | ABFPUB_000320 |

Strains and plasmids used in this study are available upon request through strain registries of the Agile BioFoundry (<http://public-registry.agilebiofoundry.org/>) and the Joint BioEnergy Institute (<https://public-registry.jbei.org/>), designated by ABF and JBx, respectively. Unless indicated, sequences are from *R. toruloides*, with the exception of antibiotic resistance genes. *GAPDH*, glyceraldehyde 3-phosphate dehydrogenase; BIS, α-bisabolene synthase from *Abies grandis* (NCBI Accession Number, O81086), *NOS*, nopaline synthase from *A. tumefaciens* (MK078637); *ANT*, adenine nucleotide translocase; SpHMGR, HMGR from *Silicibacter pomeroyi* (WP_011241944); SKP1, S-phase kinase-associated protein 1 from *R. toruloides* (PRQ77980); McMK, MK from *Methanosaeta concilii* (WP_013720012); DUF, domain of unknown function from *R. toruloides* (PRQ75822); SpPMK, PMK from *Streptococcus pneumoniae* (WP_044791288); *TEF1*, translational elongation factor; *HYP3*, 1,8-cineole synthase from *Hypoxylon sp. E7406B* (AHY23922); Gg*FPS*, FPP synthase from *Gallus gallus* (P08836.2); DaHMGR, HMGR from *Delftia acidovorans* (WP_099752490); ScPMK, PMK from *S. cerevisiae* (AJS65138); Codon optimization was performed by Genscript, using a *R. toruloides* codon table, except where indicated by HC (high-CAI method). In the strain genotype description, a comma indicates sequential insertion (stacking) while an underscore indicates that the cassettes are on the same construct.


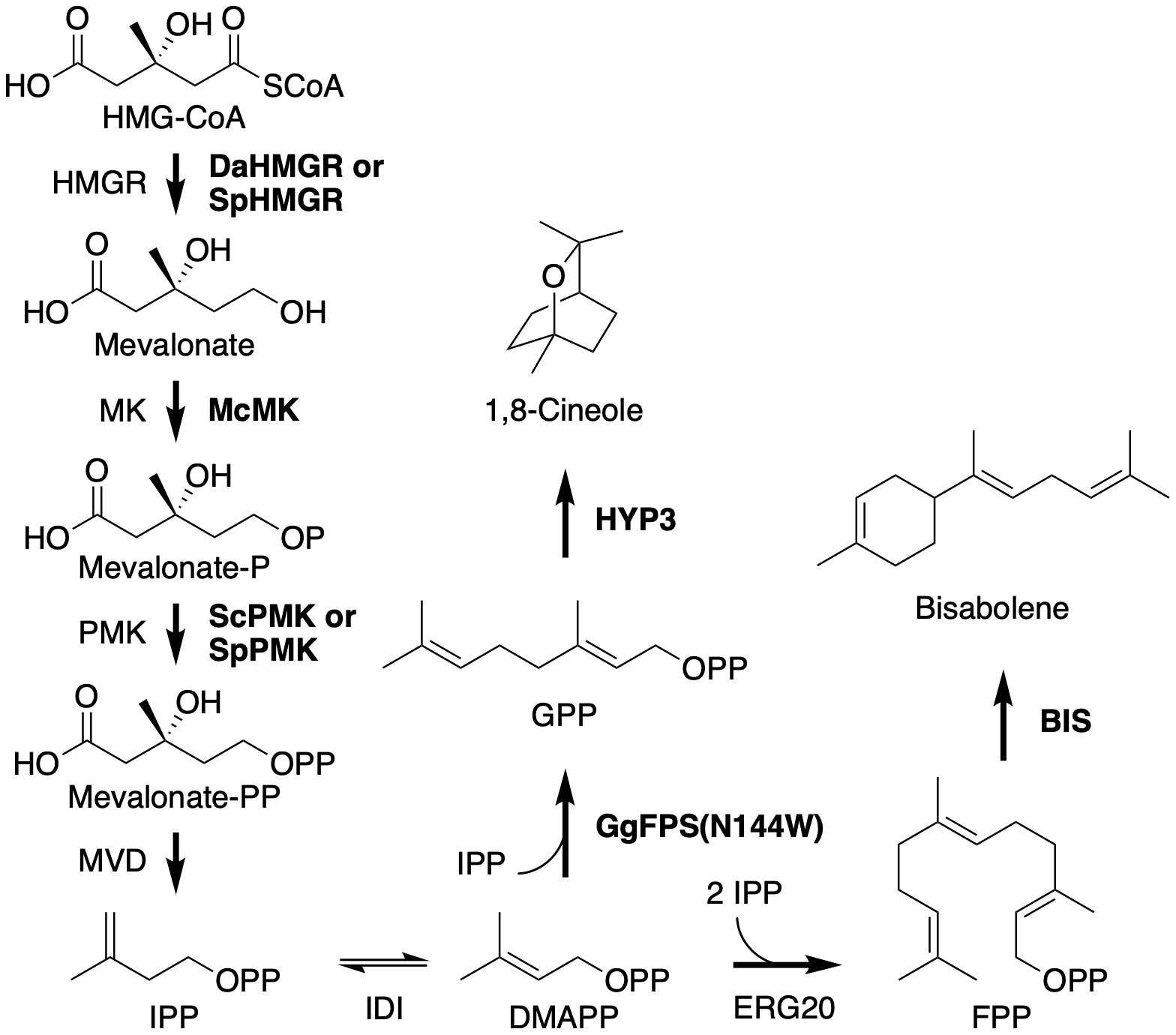


**Fig. S5** Overview of the key engineering strategies used to produce α-bisabolene and 1,8-cineole in *R. toruloides*. Heterologous enzymes expressed in *R. toruloides* in this study are highlighted in bold. HMGR, 3-hydroxy-3-methylglutaryl-CoA reductase; DaHMGR, HMGR from *Delftia acidovorans*; SpHMGR, HMGR from *Silicibacter pomeroyi*; MK, mevalonate kinase; McMK, MK from *Methanosaeta concilii*; PMK, phosphomevalonate kinase; ScPMK, PMK from *S. cerevisiae*; SpPMK, PMK from *Streptococcus pneumoniae*; MVD, mevalonate diphosphate decarboxylase; IDI, IPP/DMAPP isomerase; ERG20, FPP synthase; GgFPS(N144W), a mutant of FPP synthase from *Gallus gallus* that generates GPP; HYP3, 1,8-cineole synthase from *Hypoxylon sp. E7406B*; BIS, α-bisabolene synthase from *Abies grandis*.
